# Feasibility study of intelligent autonomous determination of the bladder voiding need to treat bedwetting using ultrasound and smartphone ML techniques: Intelligent autonomous treatment of bedwetting

**DOI:** 10.1007/s11517-018-1942-9

**Published:** 2018-12-26

**Authors:** Kaya Kuru, Darren Ansell, Martin Jones, Christian De Goede, Peter Leather

**Affiliations:** 1 School of Engineering, University of Central Lancashire, Fylde Rd, Preston, PR1 2HE UK; 2 University of Central Lancashire, Preston, UK; 3 Royal Preston Hospital, Preston, UK

**Keywords:** Ultrasound physics, Machine Learning, Bladder volume determination, Nocturnal enuresis

## Abstract

Unsatisfactory cure rates for the treatment of nocturnal enuresis (NE), i.e. bed-wetting, have led to the need to explore alternative modalities. New treatment methods that focus on preventing enuretic episodes by means of a pre-void alerting system could improve outcomes for children with NE in many aspects. No such technology exists currently to monitor the bladder to alarm before bed-wetting. The aim of this study is to carry out the feasibility of building, refining and evaluating a new, safe, comfortable and non-invasive wearable autonomous intelligent electronic device to monitor the bladder using a single-element low-powered low-frequency ultrasound with the help of Machine Learning techniques and to treat NE by warning the patient at the pre-void stage, enhancing quality of life for these children starting from the first use. The sensitivity and specificity values are 0.89 and 0.93 respectively for determining imminent voiding need. The results indicate that customised imminent voiding need based on the expansion of the bladder can be determined by applying a single-element transducer on a bladder in intermittent manner. The acquired results can be improved further with a comfortable non-invasive device by adding several more features to the current features employed in this pilot study.

**Electronic supplementary material:**

The online version of this article (10.1007/s11517-018-1942-9) contains supplementary material, which is available to authorized users.

**Graphical Abstract:**
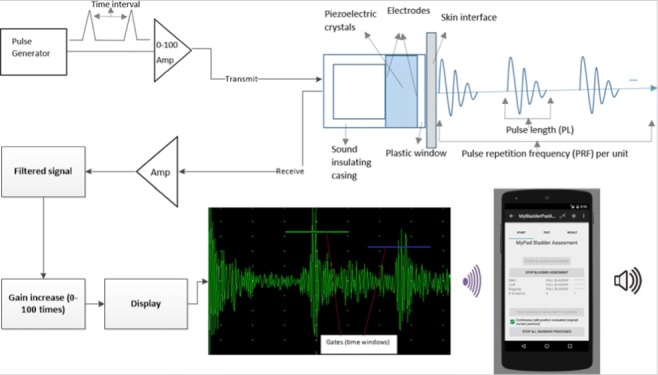
Ultrasound device design: echoed US pulses reflected from the bladder and related tissues around the bladder is detected. These pulses are analysed, and an alarm is triggered when needed to treat nocturnal enuresis.

## Introduction

Nocturnal enuresis (NE), i.e. bed-wetting, is the involuntary discharge of urine at night in a child in the absence of congenital or acquired defects of the central nervous system or urinary tract. NE is a distressing condition that affects 15 to 20% of 5-year-old children, 5% of 10-year-old children and 1 to 2% of people aged 15 years and older [1, 2]. It can have a deep impact on a child or young person’s behaviour, emotional wellbeing and social life along with parental intolerance; it is also very stressful for the parents or carers [3, 4]. NE can affect normal daily routines and social activities such as sleep overs or school trips. It can also generate much more serious feelings and behaviours, such as a sense of helplessness and a lack of hope and optimism [5], feelings of being different from others, feelings of guilt and shame, humiliation, victimisation and loss of self-esteem [6, 7]. Bed-wetting causes many practical problems as well, such as constantly having to change wet sheets and bedding. It can affect staying away overnight and going on holiday or trips. Bed wetting can affect sleep patterns and often it causes frustration and exhaustion.

The treatment of bed-wetting has a positive effect on the self-esteem of children and young people [8, 9]. There are a number of non-invasive products in the market to support the monitoring, diagnosis and treatment of bed-wetting. These products can be categorised as (i) bed-wetting alarm, (ii) pad and bell alarm and (iii) bladder scanners. A bed- wetting alarm is a mini or body-worn sensor normally worn in the pyjamas or pants. The sensor is linked to an alarm (bell or vibration alarm). If the sensor gets wet, it immediately activates the alarm. This design means the user has started voiding before the alarm is activated. The pad and bell is similar but the sensor pad is placed under the child. The alarm is activated as soon as the sensor in the mat is wet; again, the user has to have started voiding before the alarm is activated. As such, the alarm solutions on the market at present monitor and measure wetness, and our search has found no products that can predict a pre-void occurrence even though there have been several attempts to build a pre-void alarm device [10, 11]. The approaches, findings and results in our study have been compared to those in these studies in the “Discussion” section. There are a number of hand-held portable bladder scanners in the market specifically designed to capture 2D and 3D images non-autonomously. These images are then used to measure bladder volume for the purposes of diagnosing urological conditions, bladder monitoring and bladder volume management but not for the treatment/management of enuresis. These devices are for use primarily in hospital settings by physicians, clinical technicians, or nursing staff in a way of scanning the hypo-gastric region.

The National Institute for Health and Care Excellence (NICE) recommends a moisture detection alarm as a first-line treatment for a period ranging from 4 weeks to 3 months depending on the dry nights. It is important to realise that with this type of alarm, children will still wet the bed whilst learning to become dry. Medication in the form of desmopressin is an alternative and may be considered if the child, parents or carers do not want to use an alarm or are desperate to have dry nights; sometimes, a combination treatment with desmopressin and an enuresis alarm is offered; tricyclic antidepressants may be offered by an expert if all approaches above are not successful; bed-wetting may recur after being treated successfully (14 consecutive dry nights) by either moisture detection alarm or medicine [12]. Dry bed training is not recommended because there is very-poor-quality evidence to support its use and because, as part of dry bed training, the child may be punished [12]. Furthermore, many families consider the use of complementary and/or alternative medicine (CAM) such as acupuncture and hypnotherapy as a treatment option when conventional treatment fails or in order to avoid drug or other treatments [4]. There is very little evidence about the efficacy of CAM treatments, but the use of CAM is widespread and increasing across the developed world [4] based on the unsuccessful treatment results of the current methods. The cost of existing moisture detection alarms varies, but typically range from *£*80–*£*125 [13]. A 3-month treatment with Desmotabs costs approximately *£*102 at the lower dose of 0.2 mg daily and *£*204 at the higher dose of 0.4 mg daily [13]. The cost of a 3-month combination treatment of alarm and Desmotabs is approximately *£*177 at the lower dose of 0.2 mg daily and *£*278 at the higher dose of 0.4 mg daily [4]. The cost of using alarms along with other treatment approaches is mentioned in [14]: other costs included expenditure on washing and drying, extra bed clothes, underwear, pyjamas and mattresses as well as travel costs to consultations; indirect costs included time spent performing extra housework and consultation visits that prevented the carer from pursuing other activities. Treatment of NE by NHS for 6 months typically costs an extra amount of *£*400 for consultation with a specialist (*£*80 per consultation). This cost may multiply over several years for children with ongoing enuresis. It was estimated that recurrent NE costs a family *£*2,160 in extra washing, bedding, bedding protection and night clothes [15]. To summarise, total annual economic cost of NE treatment per child (excluding the costs of clinician training to deliver treatment, decrease of work/school productivity etc.) is around *£*3000. The cost is doubled or trippled where there are two or more children in a family, which is common for the families that volunteer in our patient and public involvement (PPI) phases of the project.

The use of alarms for management of NE has been in practice for just over a century [16, 17]. Whilst technology has advanced, the basic principle of the approach has remained relatively constant, alerting the child at the point of voiding, when the wetting is happening or has happened, which results in extra laundry with its costs, and interrupted family sleep patterns which can influence class room and work productivity the following day.

The initial request for innovation in this area originated from clinicians, parents and children who were unsatisfied with the performance of traditional urinary post-void moisture alarms to treat NE. The main objective of this study is to develop an effective dry alarm to treat NE and manage the bed-wetting, which would be more acceptable than currently available moisture alarms until the child has learned to control the bladder. In this manner, this study was developed to explore whether existing technologies could be synchronised, enhanced and modulated to form an intelligent alarm system that could provide a pre-void warning, minimising bed-wetting, reaching stable dryness through learning bladder control and enhancing quality of life for sufferers. This study analyses the feasibility and viability of such a system.

The data samples in our study have been analysed using three ML techniques that are Sequential Minimal Optimization (SMO), Linear Regression (LR) and Ensemble Bagging (EB) meta learning algorithms. SMO is a technique built for solving the quadratic programming (QP) problem that arises during the training of support vector machines [18]. LR is one of the most commonly used statistical method that is generally used for prediction by which a regression analysis is employed to create a mathematical model that can be used to predict the values of a dependent variable based upon the values of an independent variable using the Akaike criterion [19, 20]. EB chooses separate samples of the training dataset of the same size at random and creates a model for each independent training sample using several techniques; the results of these multiple models are then combined to reduce variance using averaged or majority voting regarding equal weight.

The organisation of the manuscript is as follows: first, the background of the study, hardware employed, data collection phase, application, settings, training, and testing and validation are presented in the “Methods” section; second the results are presented followed by the “Discussion” and “Conclusion” sections.

## Methods

### Background

We created a reference group at Alder Hey comprising of six children who were suffering from NE, and their parents. The group shared their experience of living with NE and what a negative effect it had on their lives. We met with the group to consult with them on the outline ideas and plans for our pre-void warning alarm device. The group shared design and operation requirements with the team. Factors such as ease of use, ergonomics, functionality and target price point were identified. The reference group gave critical feedback at key stages such as reviewing design concepts. The group’s feedback and design requirements enabled us to determine the key features of a good device, refine the initial design specifications and specify the functionality of the electronics. After an investigation of different sensing methods, including low cost EMG and Ultrasound (US) technology, single channel US was found to be safe and to provide the most promising of the sensor technologies and the level of data required to issue a pre-void warning. In this manner, we planned to develop an effective pre-void alarm to manage the NE problem better than the existing methods using single-element US and Machine Learning (ML) approaches.

Before describing our techniques and approaches, we would like to explore the characteristics of the urinary bladder along with ultrasound physics in summary. Then, the techniques, methods and approaches employed in the study along with the experiments are explored following this summary.

#### Urinary bladder and ultrasound physics

For humans, in supine position, the anterior bladder wall lies at an average depth of about 4 cm, which also depends on the bladder filling stage [21]. In most situations, a full bladder will be closer to the abdominal wall than an empty bladder [21]. In males, the empty bladder has a triangular or prism shape, and its largest diameter is oriented perpendicular to the abdominal wall; in females, the bladder is located lower in the pelvis and the prism-like shape is a bit more flattened [21] (Fig. 11 in Supplementary materials). The pubic bone between the bladder and the abdomen wall makes it difficult to observe the bladder using a non-invasive approach, as US cannot penetrate bone. In children, the bladder lies at a higher level than in adults, above the pubic bone [21], which makes it easier to analyse the bladder compared to adults. In most cases its shape resembles an ellipsoid, oriented roughly parallel to the abdominal wall. When the bladder fills up, it first distends in depth (towards the spine) and then expand in height into the abdominal cavity [21], which makes it easier to analyse the bladder as it fills. The lowest part of the bladder in standing position, the base, will remain behind the pubic bone. Orientation of the transducer with an appropriate angle is required to send US beams into the bladder above the pubic bone. The normal capacity of the bladder is 400 to 600 ml in adults [22] and the maximum amount of urine an adult bladder can hold ranges from 500 to 1000 ml and depends on the individual [21]. The capacity of the bladder for the children between 6 and 14 ranges from 240 to 400. The urge to void usually starts at the third state of the bladder [22], (i.e., three quarters full, 3/4). During urination, the bladder muscles contract, and two sphincters (valves) open to allow urine to flow out [22]. These muscles are controlled by spinal nerves [23]. The internal sphincter is under autonomic control, whilst the external sphincter is under voluntary control. When any of these actions occur involuntarily, leakage can result [23]. To conclude, the characteristics of the bladder along with its environment change according to the sex, age, body mass index (BMI), amount of urine inside and position of the body. Therefore, there is no specific definition of the bladder shape.

**Fig. 1:**
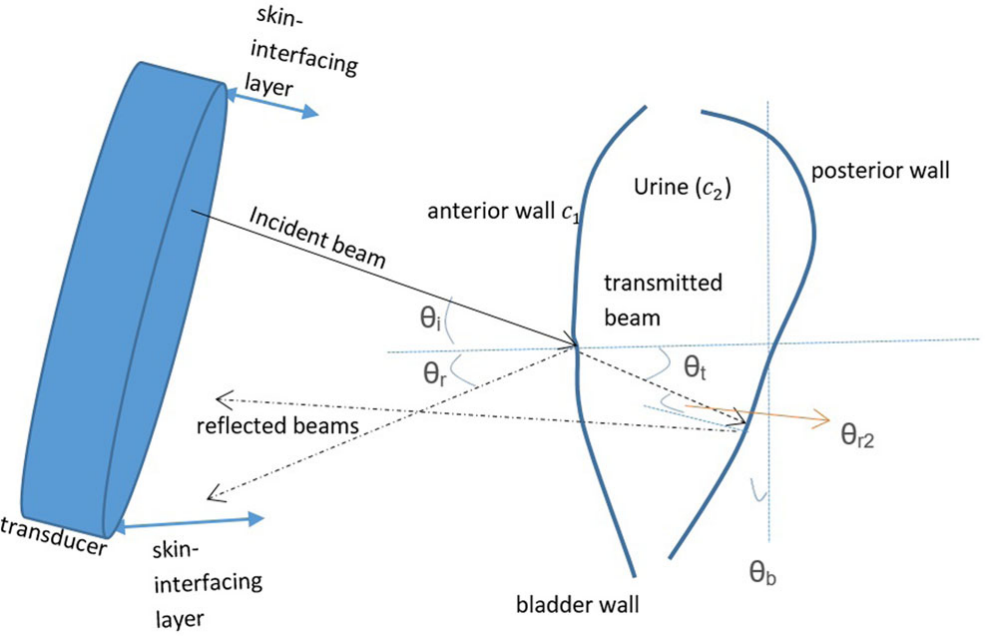
Interaction of US beams and the bladder

**Fig. 2:**
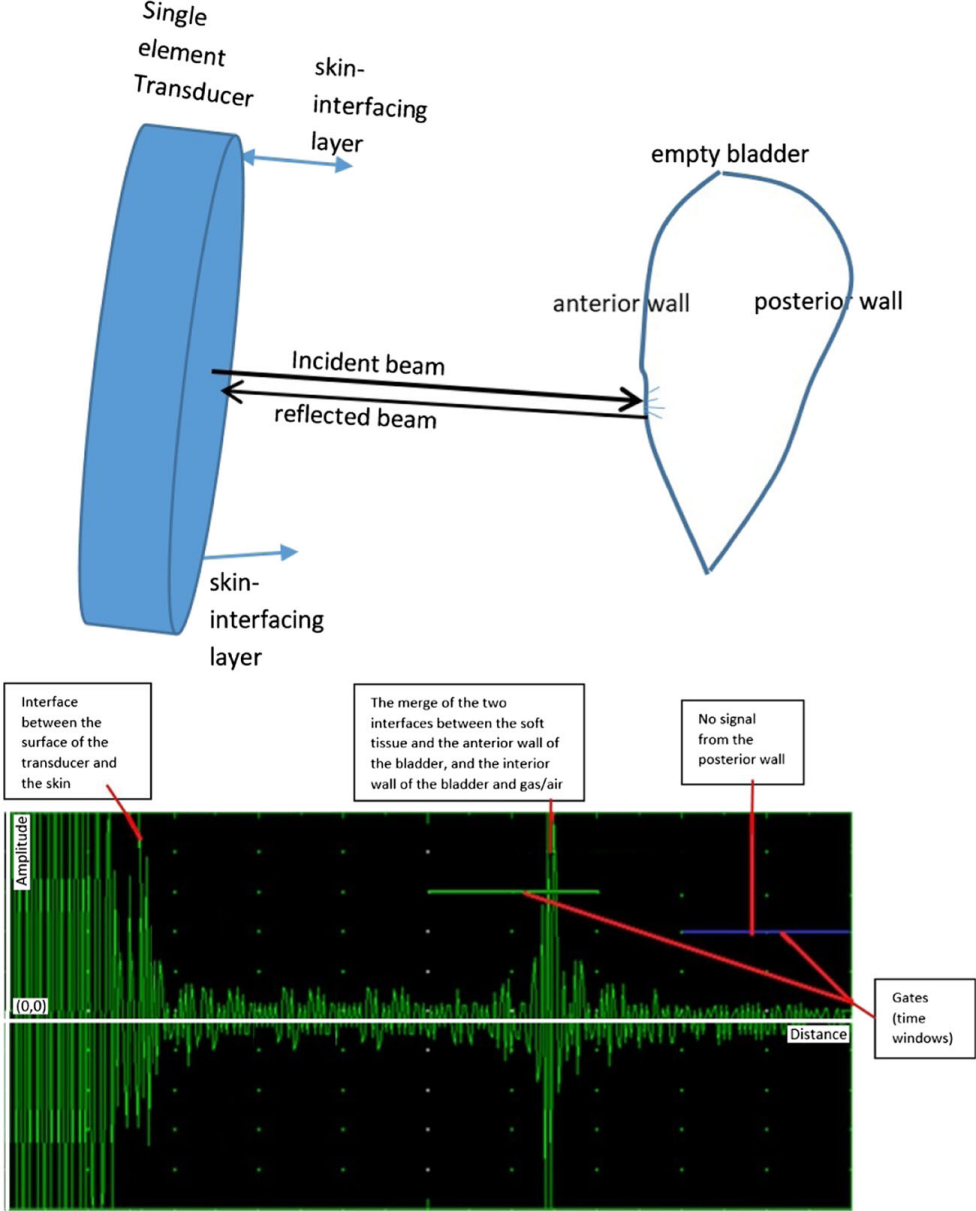
Interaction of US beams and the bladder with respect to an empty bladder: a single-element US transducer and respective propagation and attenuation signals; the pulsed signal is almost completely reflected (99%) and the remaining propagated signal after the anterior wall of the bladder is scattered in all directions in a non-uniform manner due to emptiness within the bladder

**Fig. 3:**
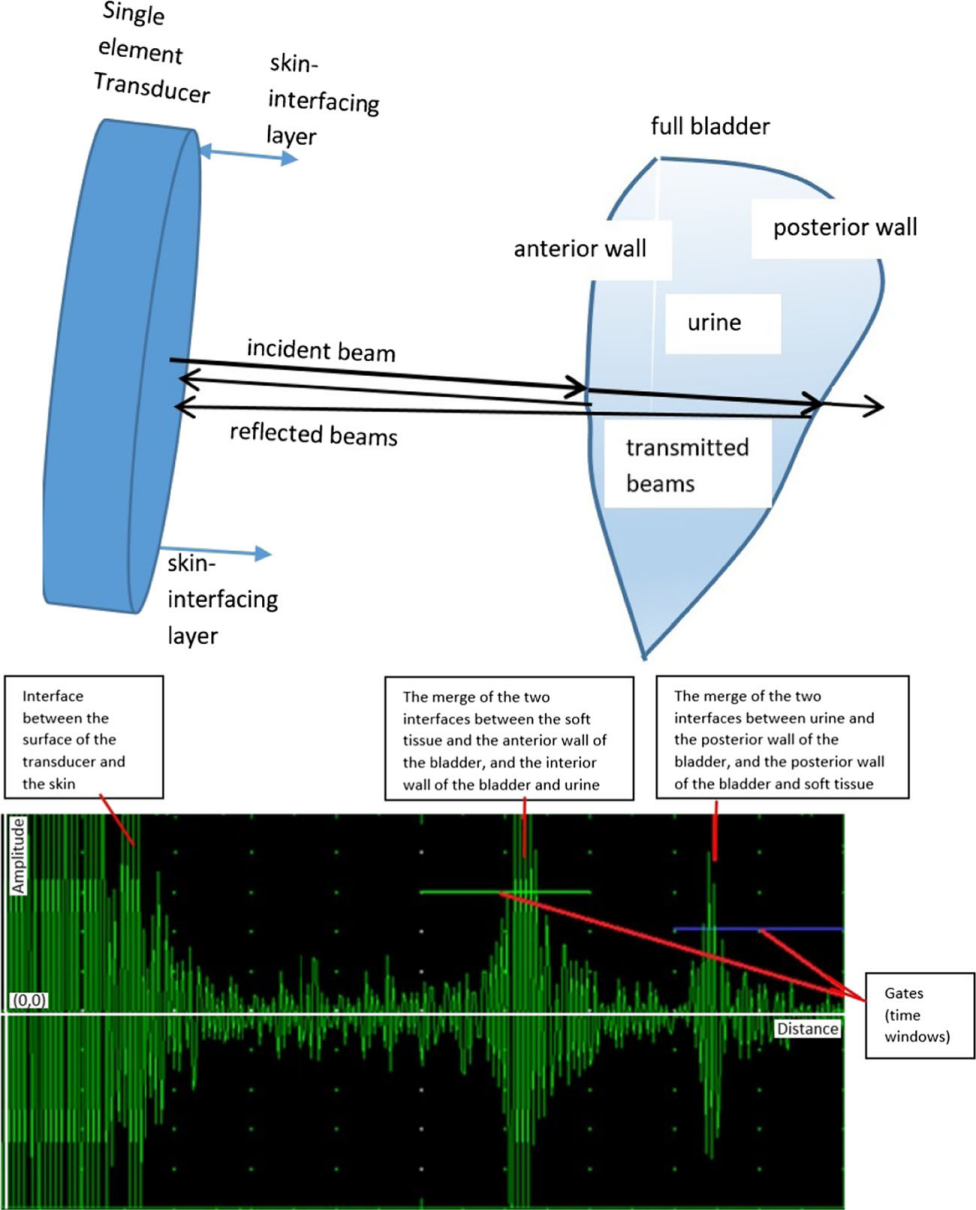
Interaction of US beams and the bladder with respect to a full bladder: single-element US transducer and respective propagation and attenuation signals where the reflection is 5% and propagation signals after the anterior wall of the bladder do not lose their strength because the urine inside the bladder causes little attenuation

The relationship of US beams and the bladder in broader perspective is illustrated in Fig. 1. This relationship is depicted with respect to empty and full bladder in Figs. 2 and 3 respectively. The reflection of the pulses from the anterior wall of the bladder where the bladder is empty is 99.9% [24], which illustrates the difficulty in transmitting emitted pulses beyond the distal side of the bladder. It is 5% [24] (Table 3 in the Supplementary materials) where there is urine by which an emitted signal can reach the posterior wall without any difficulty. Bladder wall thickness (BWT) starts to decrease above a bladder filling of 50 ml [25] ranging from ≈3 mm for a full bladder to ≈5 mm for an empty bladder [26, 27]. Measuring the movement of the front and back wall of the bladder along with the thickness of the walls is a strong indication of the level of expansion and the amount of urine inside the bladder. The detailed technical information about the basics of US physics regarding bladder and safe use of ultrasound in medicine with respect to human body, particularly bladder related to our approaches and techniques has been explored in the Supplementary materials.

### Hardware and parameters selected for the safe use of ultrasound

In this study, we follow ALARA (As Low As Reasonably Achievable) principles and have ensured that the exposure time and intensity of US applied in our approach provides a safe environment for the children with NE. In this manner, both the intensity and exposure time are reduced and as concluded in the Supplementary materials, with single-element low-frequency US along with short exposure time and low pulse repetition frequency there is no adverse biological effect.

**Fig. 4:**
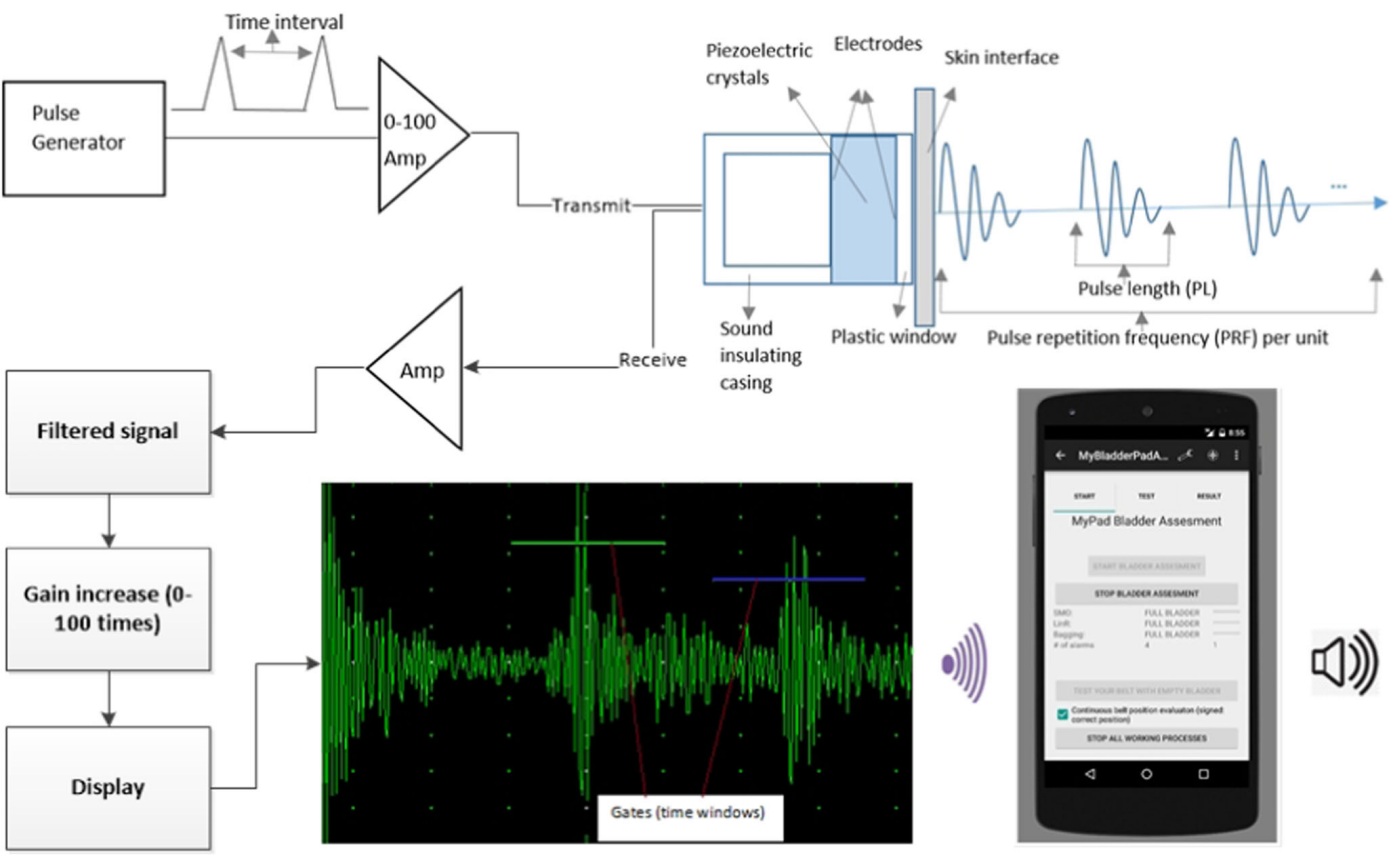
Ultrasound Design: the echoed US pulses reflected from the bladder and related tissues around the bladder is detected using a skin-interfacing gel between the transducer and the skin

**Fig. 5:**
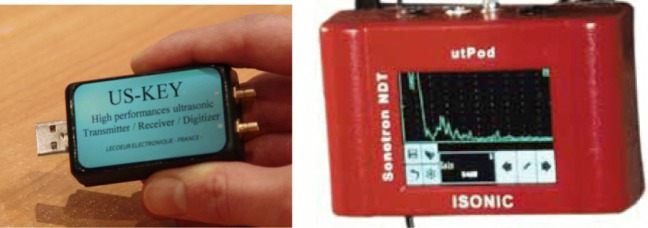
US-Key ultrasonic Transmitter/Receiver/Digitizer (Leoceur Electronique) (left) by which data can be acquired using a computing device and ISONIC utPOD device (right) by which data can be acquired using both itself and a computing device

We employ A-mode use of US in our study using single element transducer without scanning, and we do not aim to get an image from the bladder. Therefore, we do not need to apply US beams a long time nor with big intensity values (W/cm^2^). The components of the designed US are depicted in Fig. 4. A low-frequency, single-element transducer with 2.2 MHz and a diameter of 1.5 cm and a static matching layer attached to ultrasonic transmitter/receiver/digitiser devices shown in Fig. 5 is employed to transmit US pulses and receive echoed pulses with a transmitter voltage of 80V. An acoustic gel was used between the transducer and skin to transmit the pulses. ROI with this transducer is 20 cm in human body using a US velocity of 1550 as shown in Table 1. The beams were emitted in intermittent manner (duration < 25 ns). In other words, Pulse Repletion Time (PRT), the elapsed time from the beginning of one pulse to the beginning of the next pulse, was long big enough not to cause any thermal effect in addition to keeping the exposure time in milliseconds using 2-KHz pulse repetition frequency to minimise the signal emission time as low as possible. An intensity value (ISPTA) of 18.7 mW/cm^2^ was detected by AIUM for B-scan imaging [24] and our A-mode with no scanning and no imaging generates less intensity than this value, which is far less than 100 mW/cm^2^ recognised by the International Electrotechnical Commission (IEC) [28], AIUM and WFUMB as safe and well below the maximum limit, 720 mW/cm^2^, particularly for abdominal use, not causing any bioeffects. We employ a receiver gain, 80, to make the acquired signal more distinctive during the postprocessing of the echoed pulses to get the most information at the lowest output power. Therefore, our US application does not increase any temperature in the body even though it is allowed an amount less than 1° [29] by international regulations, causing no adverse bioeffects.

**Table 1:** Depth of US penetration in the body with respect to frequency

Frequency (MHz)	1	2	3	5	10	20
Penetration (cm)	40	20	13	8	4	2

At an ultrasonic frequency of 1 MHz, an average attenuation coefficient for soft tissue is approximately 0.7 dB/cm, whereas at 2 MHz, it is 1.4 dB/cm [24]; thus, the attenuation coefficient is directly related to frequency; increase one and the other increases. In this manner, Time Gain Control function was employed to adjust the amplitudes as a function of time by increasing the receiver gain with respect to attenuation as illustrated in the Supplementary materials (Fig. 2) to compensate for the effects of pulse absorption in media. The attenuation in the urine is very small (i.e., 0.00014 where c = 1551) when compared to the tissue, which makes the amplitude acquired at the posterior wall bigger where same TGC function is applied as a function of the attenuation in soft tissue (e.g., output images in Fig. 14 in the Supplementary materials) where the bladder is full with urine.

### Data collection

We aim to analyse a depth ranging from 4 cm to 20 cm where the bladder is located using a low-frequency transducer (i.e., 2.2 MHz) as explained above. The shape of the bladder changes significantly among the morphology groups (i.e., BMI, sex, age groups). Therefore, we tested our techniques on different phantoms that have different shapes to ensure that our techniques and approaches can detect available echoed pulses and work on any bladder in different shapes. The time delay between transmitting a pulse and detecting an echo signal is measured to determine the distance between the transducer and significant interfaces (*T* = 2*D*/*c* where *c* is the speed of the sound in the media, *D* is the depth of the interface and *T* is time delay to get an echoed pulse).

Our technique measures 3-dimensionality using single-element probes based on the features acquired (e.g., distance between two bladder walls, different acquired echoed pulses from the walls with respect to expansion/contraction of bladder walls (i.e., linear relationship between bladder fullness and BWT: the upper limits are ≈3 mm and ≈5 mm for a full or empty bladder respectively [26, 27]). All signal values in ROI of 20 cm as shown in the images in the Supplementary materials are analysed to reveal the distinctive features. Distinctive main features within the acquired echoed pulses utilised in ML techniques with respect to two gates^1^ shown in Figs. 2 and 3 are as follows:

- T(A):Time of flight: millisecond of an echo matching with the first gate (time window).
- T(B):Time of flight: millisecond of an echo matching with the second gate (time window).
- s(A): Distance from the transducer in millimetres of an echo matching with the first gate.
- s(A) = 1/2*T(B)*(US velocity) where US velocity is between 1436 (for fat) and 1550 (for muscle) for human body based on the body mass index (BMI).
- s(B): Distance from the transducer in millimetres of an echo matching with the second gate.
- s(B) = 1/2* *T*(*B*)*(US velocity) where US velocity is between 1436 (for fat) and 1550 (for muscle) for human body based on the body mass index (BMI) until first signal, and 1551 (for urine) between the first and second signal.
- Δ*T* = *T*(*B*) − *T*(*A*)
- Δ*s* = *s*(*B*) − *s*(*A*)
- H(A): Amplitude: % of A-Scan height of an echo matching with the first gate.
- H(B): Amplitude: % of A-Scan height of an echo matching with the second gate.
- V(A): Amplitude (relative signal level; db (decibel) of an echo matching with the first gate with respect to a threshold.
- V(A) = 20*log10(H(A)/thresholdA) where V(A) is relative signal level (dB) at the first gate,
- V(B): Amplitude: (relative signal level); db of an echo matching with the second gate with respect to a threshold.
- V(B) = 20*log10(H(B)/thresholdB)
- Δ*V* = *V* (*B*) − *V* (*A*)

#### Data collection from volunteers

The data samples were collected from two volunteers in their homes with their families. The volunteers were male and first volunteer is 6 years old with a BMI, 18.9.^2^ The second volunteer is 9 years old with a BMI, 23.4. The details of the data samples are presented in Table 2. Data samples were acquired by applying a single-element transducer attached to the ultrasonic transmitter/receiver/digitiser devices shown in Fig. 5 on the abdomen using the anterior sagittal section on the position of supine. The transducer with a 15° with respect to the horizontal axis was placed in the hypo-gastric region using tapes with a ratio of 2:1 between the navel and pubic bone, approximately 10 cm below the navel and 5 cm above the pubic bone to locate on a flat surface above the pubic bone right across the bladder. The same location was marked with a circle and all data was acquired from this designated location. First, the children emptied their bladder and the instant data was collected from the empty bladder. And then, the data was systematically recorded from the different urine volumes of the bladder in intermittent manner ranging from 5 to 30 min intervals as the volume in the bladder increased based on the liquid (i.e., juice or water) consumed before and during the data collection phase. 10 to 20 data samples were acquired during each US application regarding the cooperation of the children. The children did not drink any liquid during the hour prior to the data collection phase from the empty bladder to correlate the intake of fluid and the extension of the bladder in terms of the time. The children were asked to drink liquid as much as possible during detection of the data samples to shorten the duration of the data collection time. They were asked not to pass urine as long as possible to determine both the full state and especially the point where they started having the urge to go. The duration of collecting data between empty and full bladder ranged from 140 min to 170 min depending on the children and the amount of liquid they consumed. The children watched television during data acquisition and they were free during intervals to do any activity they liked to provide better cooperation. Sticking the probe to the same location and collecting data each time between intervals took 3–10 min depending on the cooperation of the children. The amount of urine was measured after voiding and compared to the expected average urine volume based on the age to ensure the full state of the bladder. The voided volumes ranged from 210 to 335 ml in our experiments. This process was repeated 5 times on different days. The total number of the acquired data samples is 3090 (i.e., 1478 from 6-year-old volunteers and 1612 from 9-year-old volunteers). The numbers of the data samples for the four status of the bladder (i.e., empty, 1/2 (half), 3/4 (three quarters), and full) are 826, 844, 740 and 680 respectively. The output examples for these status are presented in the Supplementary materials, Figs. 12, 13, 14 and 15. The summary of the data samples are presented in Table 2.

**Table 2:** Data samples collected from the volunteers

Volunteer	Days	Duration (min)	Intervals (min)	No. of observations	Liquid consumed (ml)	Urine measured (ml)	Ref bladder volume*	Samples acquired
V1	Day-1	163	15	8	350	215	240	304
V1	Day-2	157	15	7	500	225	240	270
V1	Day-3	142	15	8	500	235	240	310
V1	Day-4	168	30	4	500	255	240	284
V1	Day-5	170	30	5	500	230	240	310
V2	Day-1	155	15	9	500	330	330	330
V2	Day-2	178	15	8	500	335	330	314
V2	Day-3	160	15	8	400	310	330	340
V2	Day-4	155	30	5	500	330	330	324
V2	Day-5	164	30	6	300	315	330	304

### The application and ML techniques

**Fig. 6:**
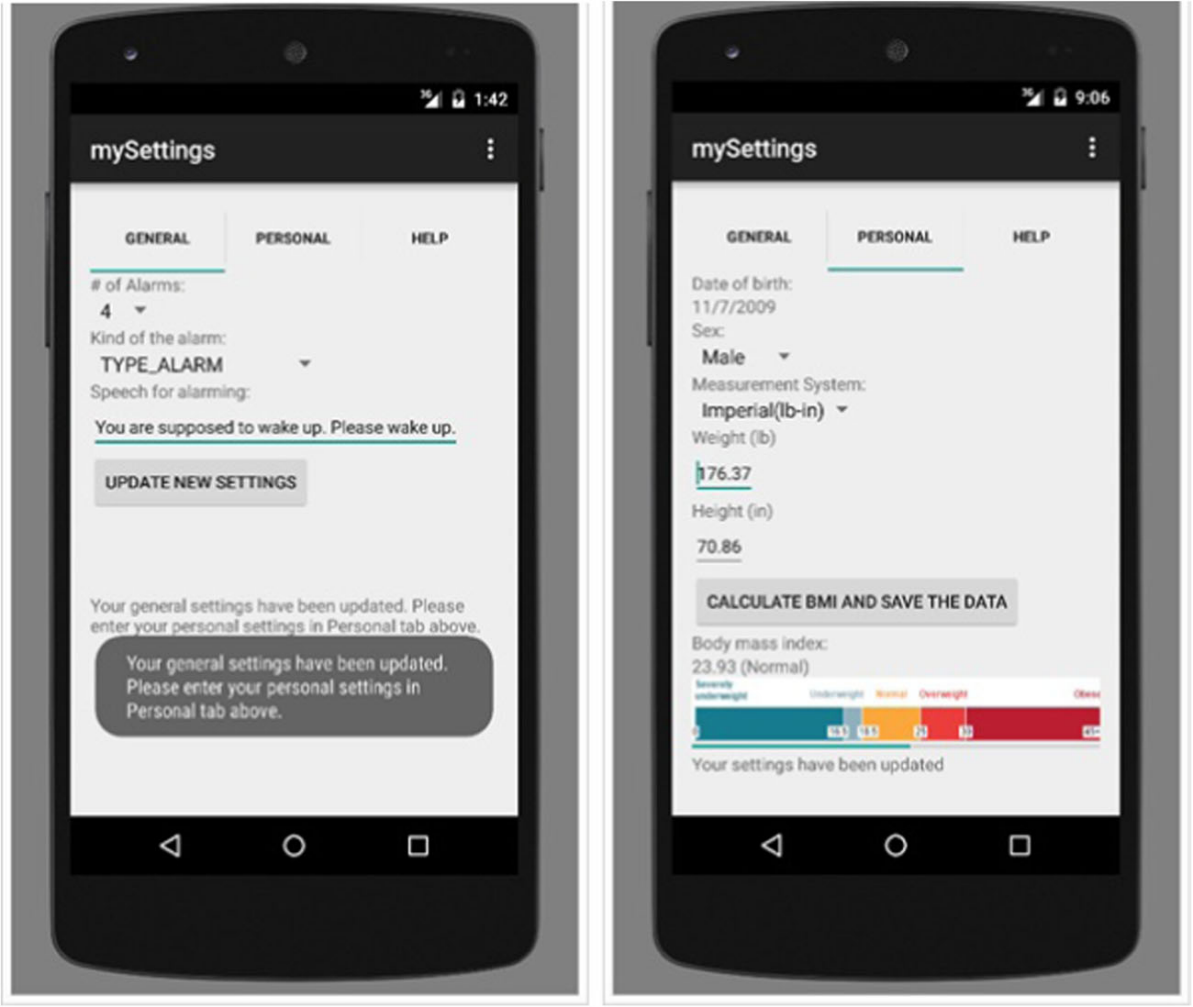
Settings: General settings and personal settings

**Fig. 7:**
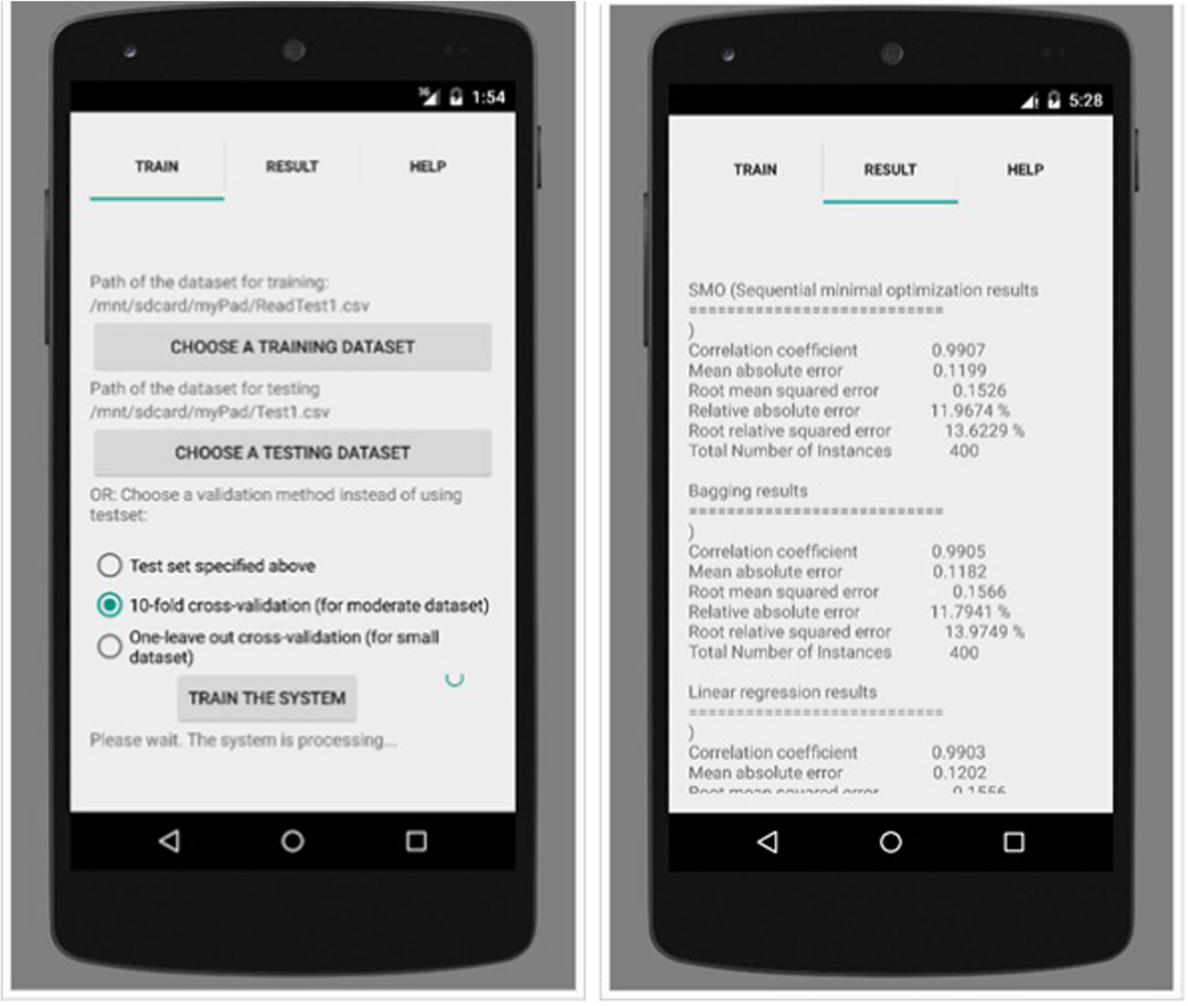
Training is aimed to be implemented at the background automatically when needed without user intervention: **a** selection of the training dataset and training approach to train the system; **b** statistical results of the three ML techniques after training using 10-fold cross-validation

**Fig. 8:**
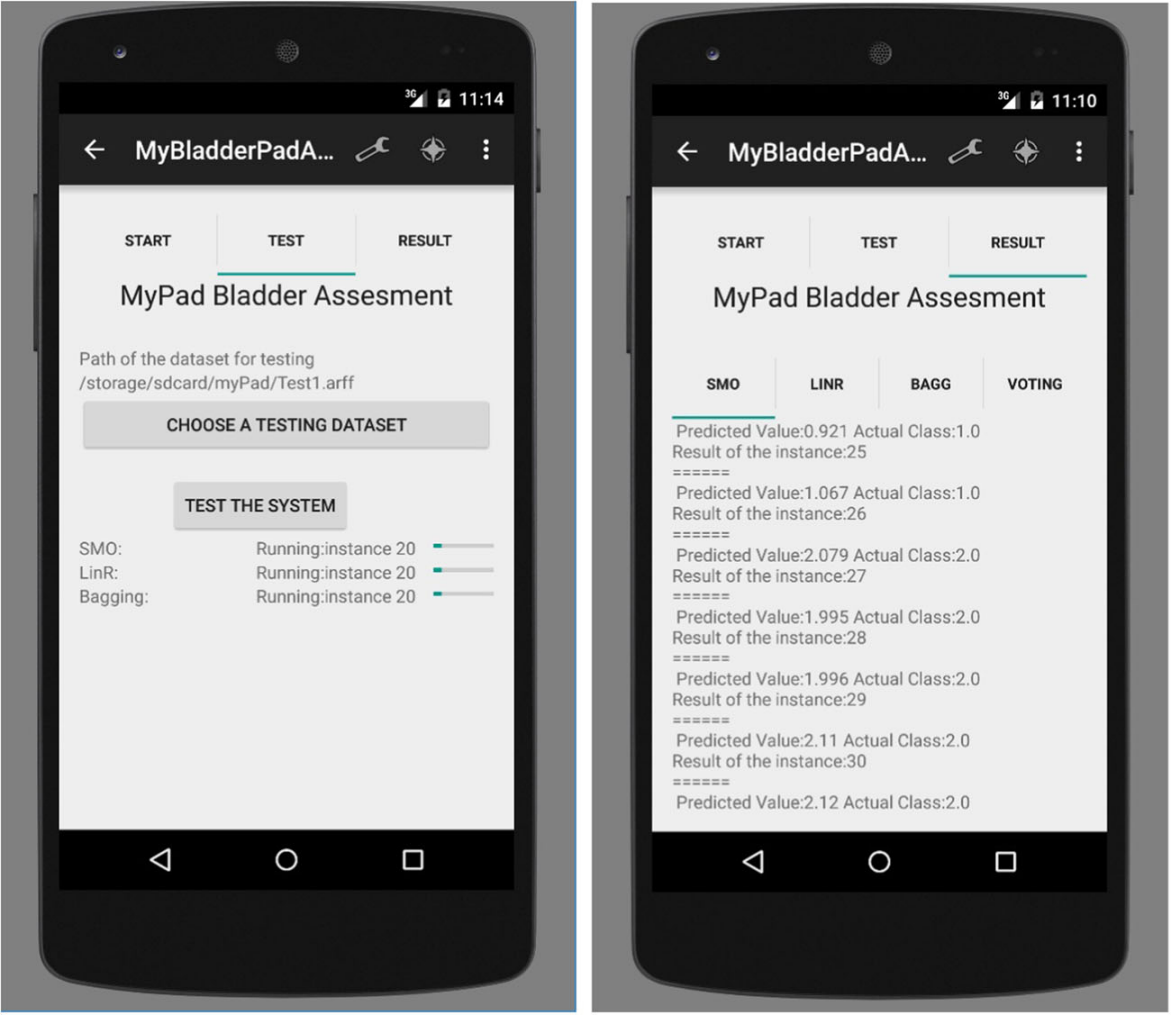
Testing: **a** selection of the test dataset (left); **b** statistical results of the three ML techniques and voting scheme (right)

A smartphone application was built using Java programming language within Android Studio Platform to analyse the distinctive features of the echoed pulses generated from the urinary bladder, urine and surrounding media. The screen-shots of this application are presented in Figs. 6, 7, 8 and 12. Its size is 9 MB and it is particularly developed for Android operating systems using multi-threaded approach in order to process several tasks at a time; it has been designed to work in any ordinary cell-phone not requiring much memory (preferably > 4GB) and CPU. The running times for specific tasks are presented in Section 2.6.

The application employs several ML techniques on the dataset using holdout, n-fold and leave-one-out cross-validation (CV) schemes depending on the number of the instances. The ML techniques that fit the dataset best are Sequential Minimal Optimization (SMO), Linear Regression (LR) and Ensemble Bagging (EB) meta learning algorithms that were tested on sample datasets and found to be the successful. These algorithms take several parameters based on the count of the instances and characteristics of the datasets and some parameters are embedded in the application as being the optimised parameters.

SMO is a technique built for solving the quadratic programming (QP) problem that arises during the training of support vector machines [18]. Training a support vector machine requires the solution of a very large QP optimisation problem. SMO breaks this large QP problem into a series of smallest possible QP problems. These small QP problems are solved analytically, which avoids using a time-consuming numerical QP optimisation as an inner loop. The amount of memory required for SMO is linear in the training set size, which allows SMO to handle very large training sets in a time efficient way. On real world sparse datasets, SMO can be 1000 times faster than SVM [18]. The second ML algorithm, LR, is one of the most commonly used statistical method that is generally used for prediction. The goal in regression analysis is to create a mathematical model that can be used to predict the values of a dependent variable based upon the values of an independent variable using the Akaike criterion [19, 20] to find the best model that fits the dataset best. The final ML algorithm, EB, chooses separate samples of the training dataset of the same size at random and creates a model for each independent training sample using several techniques. The results of these multiple models are then combined to reduce variance using averaged or majority voting regarding equal weight, which looks like our approach here in combining three ML techniques using averaged or majority voting.

The system can evaluate the dataset using 3 CV approaches as shown in Fig. 7, namely Holdout CV, k-fold CV and leave-one-out CV (LOOCV) schemes. Holdout CV split the data samples into two parts, (e.g. 50%/50%), one for training and one for testing. It is advantageous to use this scheme if there is a very big dataset to analyse. Regarding small dataset, if we use half of the data for the test set, we are only training the half of the data and we may get a poor hypothesis; if we reserve only 10% of the data for the test set, then we may, by statistical chance, get a poor estimate of the actual accuracy [30]. We can obtain more information out of the small dataset and still get an accurate estimate using a technique called k-fold CV. The idea is that each example serves double duty-as training data and testing data. First, we split the data into k equal subsets. We then perform k rounds of learning; on each round 1/k of the data is held out as a test set and the remaining examples are used as training data [31]. The average test set score of the k rounds should then be a better estimate than a single score. On the other hand, LOOCV requires longer computation time and is usually employed for small datasets. It is not advised for moderate and big datasets. In LOOCV, one instance is left out as a test instance and the system is trained using the remaining instances. This process is repeated as many times as the number of the instances in the dataset, so every data point is used as a test sample to measure its similarity to the others.

The discriminant features extracted from the datasets based on the specific characteristics of urine in the bladder, the bladder itself and surrounding tissues are processed to train the system and to determine the relative fluid level in the bladder for subsequent uses based on the trained models. SMO, LR and EB are forged to each other to determine the status of the bladder based on a voting system that uses the predictions of 3 types of bladder status classifiers produced by three well-known ML techniques mentioned above. The implementation phases of ML techniques such as training, testing and validation are explained in the following sections specific to the datasets we have.

### Settings

Several settings specific to the user should be defined in the application as displayed in Fig. 6 before starting training, testing, validation and real-time use of the application. These settings can be entered using two screen tabs, namely general and personal tabs. In the general tab, the number of alarms, type of the sound alarm and text-to-speech voice alarm are entered. In the personal tab, BMI is calculated using height and weight and standardised against age and sex dependent values. The setting of these parameters is explained in a scenario in the “Discussion” section (Fig. 14). BMI is a key element for the following phases as body habitus may affect the position of the US machine, and consequently affect the measurements. These steps are illustrated in a scenario in Section 4 (Fig. 14).

### Training

**Fig. 9:**
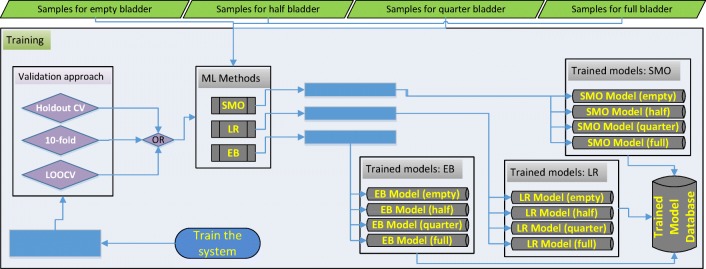
Phases in training: classifiers in four groups with respect to three ML techniques are built

Training is the learning phase in which the machine learns what a normal bladder looks like, and how it increases in volume, including where urge and maximum volume/voiding point. The screen-shot of the application for training is presented in Fig. 7. 90% of the acquired data samples (Table 2) was used for training. Based on the number of the dataset we have, the training was performed using 10-fold CV and the system was trained accordingly as mentioned above with respect to k-fold CV. Training data is used to train the SMO, LR and EB classifiers to establish the learning models. The algorithms are run in multi-threaded way by dividing code particles among multiple processors for the objective of running these code particles in a time efficient way. Thus, the required running time to establish the classifier models of the ML techniques for the training dataset ((i.e., 743, 760, 666 and 612 for empty, 1/2, 3/4 and full respectively) ranges from 600 to 800 s in a parallel processing way. The discriminant features from training data including BMI and sex, some of which are mentioned above are saved in these models for further use of determining imminent voiding. In other words, the application sets a customised warning trigger point based on the bladder expansion cycle with respect to the likelihood of an imminent voiding of the bladder by learning the bladder characteristics specific to the children using these models. The phases of training are presented in Fig. 9. The testing phase specific to changing circumstances is illustrated in a scenario in Section 4 (Fig. 15).

### Testing and validation

Testing is the phase to measure the performance of the system whereas validation is the phase to measure how well the system predicts voiding, and whether it alarms in time.

The screen-shot of the application for testing is presented in Figs. 8. 10% of the acquired data samples (i.e., 83, 84, 74 and 68 for empty, 1/2, 3/4 and full respectively) was used for testing. A voting scheme based on the results of the three classifiers is performed to determine on the current state of the bladder. The worn of the undergarment is tested with empty bladder based on the characteristics of the echoed pulses acquired from the empty bladder.

The screen-shot of the implementation for validation is depicted in Fig. 12. Final bladder status is determined by the weighted average/majority of the plurality of three distinct bladder status opinions as new instances are introduced into the system, and accordingly a pre-void alert type customised for the user (audible and/or vibrating alarm) is triggered if the level is indicating a triggering point. The alert signal may also be received by a third party’s (e.g. parent or carer) smartphone optionally to inform them to check the child.

**Fig. 10:**
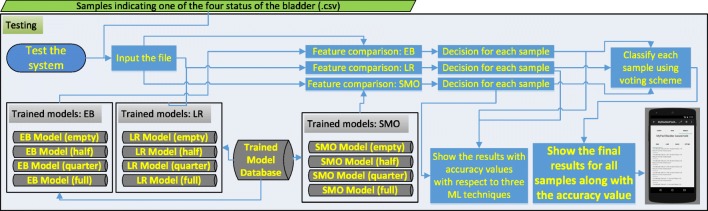
Phases in testing: classification of the samples are performed with respect to the comparison between the features of each sample and pre-trained models

**Fig. 11:**
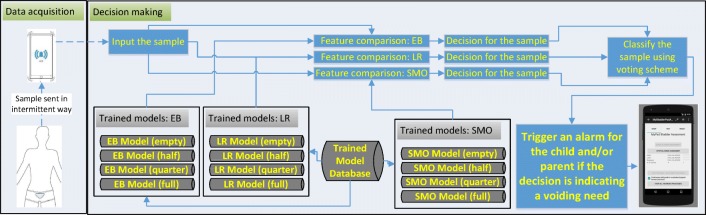
Phases in data acquisition and decision making: classification of the acquired samples are performed with respect to the comparison between the features of the sample and pre-trained models

**Fig. 12:**
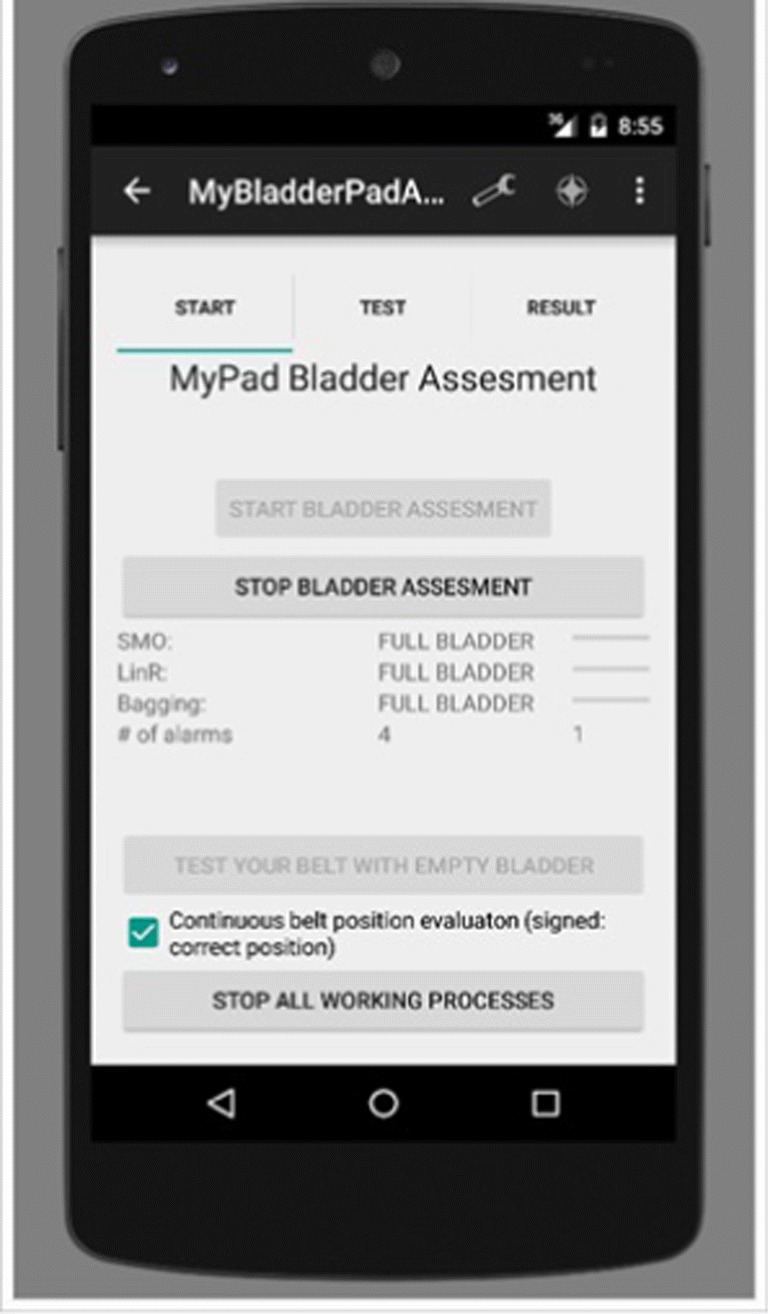
Determination of bladder status and control of the undergarment placement

The device to be developed and mentioned in the discussion and future work sections has been designed to record a voiding event through the device’s moisture sensor detecting wetness in the case of an involuntary voiding event to customise itself for the current user in terms of the triggering threshold using self-tuning features as illustrated in the scenario in the “Discussion” section. For instance, if a moisture alarm is triggered without a prior pre-void system alarm, then the triggering point of the pre-void alarm should be adjusted accordingly; if there is a prior pre-void alarm record before moisture alarm, then it means that the system is working properly, but the alarm types are not effective to wake up the child. The phases of testing and validation are presented in Figs. 10 and 11 respectively. These phases are explored in a scenario in the “Discussion” section as well (Fig. 15 and Fig. 12).

## Results

**Fig. 13:**
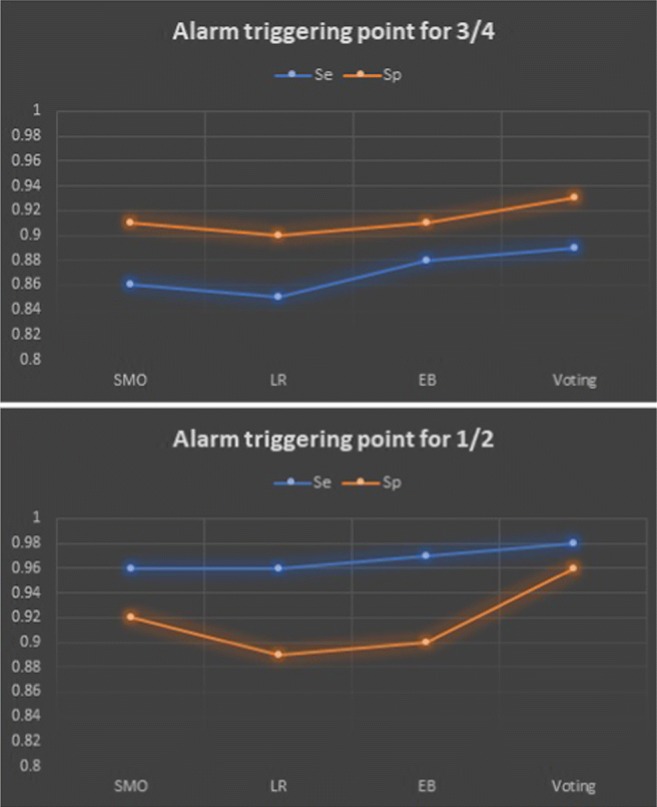
*Se* and *Sp* values with respect to the techniques

The success rates of the ML learning techniques regarding data sample classes are presented in Tables 3, 4 and 5, and the results of merging these techniques using voting scheme is displayed in Tables 6. The sensitivity and specificity values^3^ of these techniques along with the voting scheme are depicted in Table 7 and Fig. 13. The EB technique has the biggest *Se* (i.e., 0.97 for 1/2 and 0.88 for 3/4 triggering points), which is slightly increased by voting scheme (i.e., 0.98 for 1/2 and 0.89 for 3/4). SMO technique has the biggest *Sp* (i.e., 0.91 for 3/4 and 0.92 for 1/2 triggering points), which is slightly increased by voting scheme too (i.e., 0.96 for 1/2 and 0.93 for 3/4).

**Table 3:** Training results of SMO technique

SMO	Empty:743		1/2:760		3/4:666		Full:612	
Status	#	%	#	%	#	%	#	%
Empty	*681*	*91.66*	53	6.97	14	2.10	5	0.82
1/2	36	4.85	*601*	*79.08*	98	14.71	59	9.64
3/4	19	2.56	71	9.34	*438*	*65.77*	129	21.08
Full	7	0.94	35	4.61	116	17.42	*419*	*68.46*
Total	743	100	760	100	666	100	612	100

The italic numbers in the cells correspond to the correct determination of the bladder status

**Table 4:** Training results of LR technique

LR	Empty:743		1/2:760		3/4:666		Full:612	
Status	#	%	#	%	#	%	#	%
Empty	*657*	*88.43*	61	8.03	19	2.85	10	1.63
1/2	54	7.27	*586*	*77.11*	103	15.47	65	10.62
3/4	23	3.10	77	10.13	*413*	*62.01*	135	22.06
Full	9	1.21	36	4.74	131	19.67	*402*	*65.69*
Total	743	100	760	100	666	100	612	100

The italic numbers in the cells correspond to the correct determination of the bladder status

**Table 5:** Training results of EB technique

EB	Empty:743		1/2:760		3/4:666		Full:612	
Status	#	%	#	%	#	%	#	%
Empty	*665*	*89.50*	56	7.37	11	1.65	2	0.33
1/2	49	6.59	*598*	*78.68*	93	13.96	42	6.86
3/4	21	2.83	71	9.34	*426*	*63.96*	135	22.06
Full	8	1.08	35	4.61	136	20.42	*433*	*70.75*
Total	743	100	760	100	666	100	612	100

The italic numbers in the cells correspond to the correct determination of the bladder status

**Table 6:** Training results of voting scheme

Voting	Empty:743		1/2:760		3/4:666		Full:612	
Status	#	%	#	%	#	%	#	%
Empty	*712*	*95.83*	27	3.55	7	1.05	1	0.16
1/2	19	2.56	*642*	*84.47*	84	12.61	51	8.33
3/4	9	1.21	65	8.55	*463*	*69.52*	127	20.75
Full	3	0.40	26	3.42	112	16.82	*433*	*70.75*
Total	743	100	760	100	666	100	612	100

The italic numbers in the cells correspond to the correct determination of the bladder status

**Table 7:** *Se* and *Sp* values of the schemes with respect to the alarm triggering point of 3/4 and 1/2

	SMO		LR		EB		Voting	
	3/4	1/2	3/4	1/2	3/4	1/2	3/4	1/2
Se	0.86	0.96	0.85	0.96	0.88	0.97	0.89	0.98
Sp	0.91	0.92	0.9	0.89	0.91	0.9	0.93	0.96

## Discussion

**Fig. 14:**
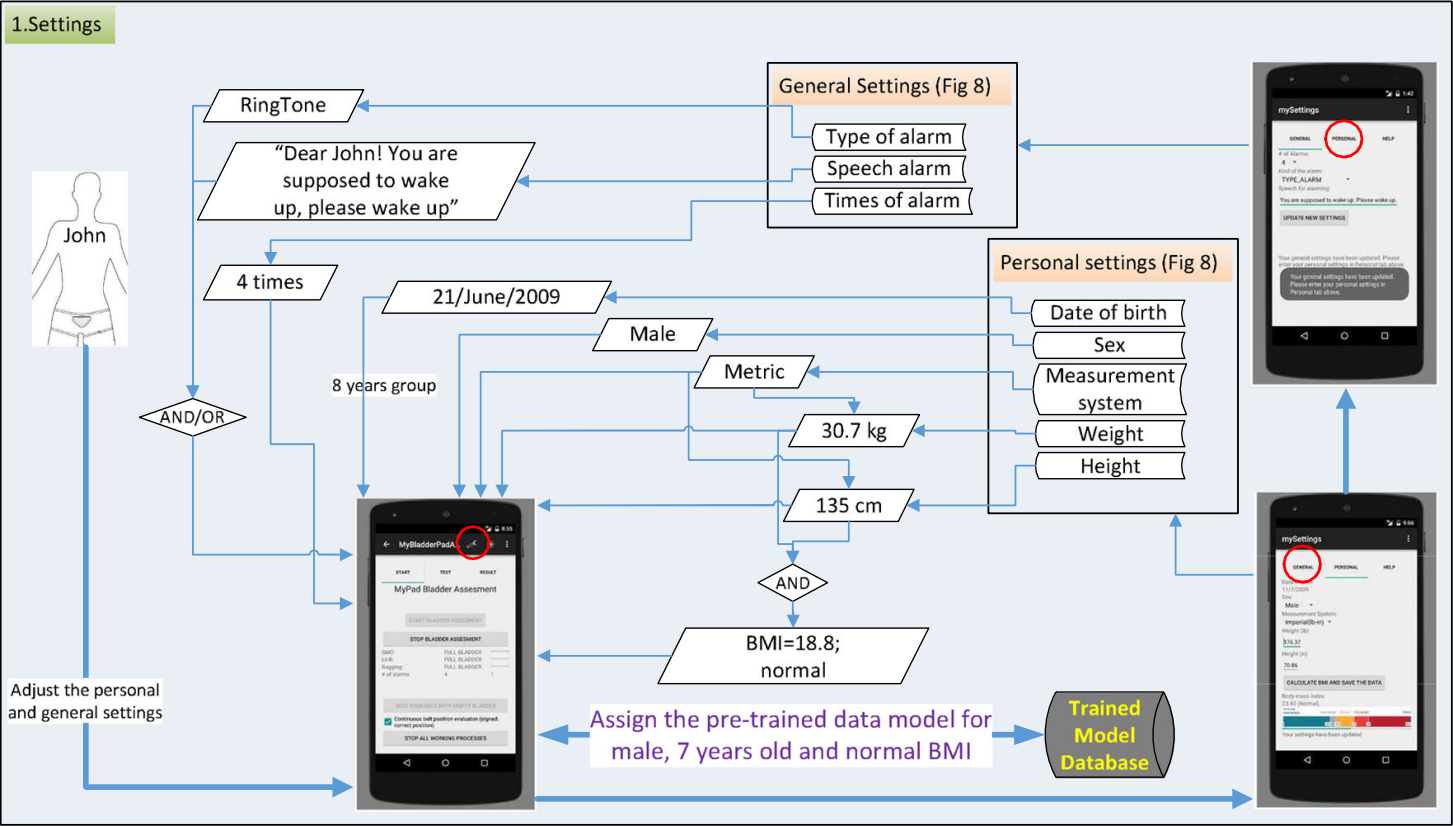
Scenario for settings

**Fig. 15:**
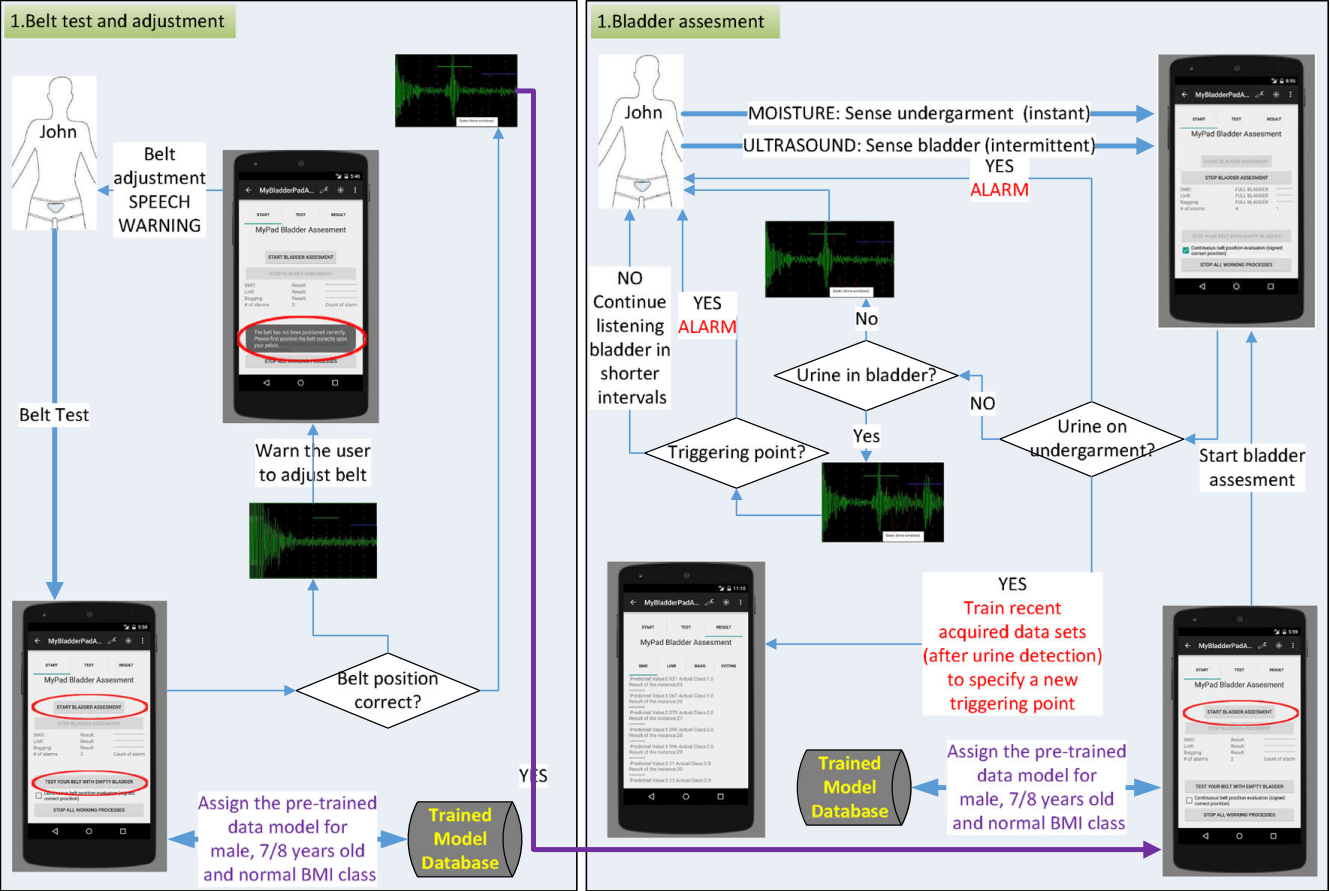
Scenario for bladder assessment

This study was carried out to explore whether existing technologies could be synchronised, enhanced and modulated to form an intelligent alarm system that could provide a pre-void warning, minimising bed-wetting, reaching stable dryness through learning bladder control and enhancing quality of life for children who wet the bed. The solution is designed to be an intelligent autonomous decision system that uses multiple methods to trigger a pre-void warning that can be customised to the user’s physical characteristics by combining several measurement attributes of the bladder when it is full, expanded or empty. In this manner, we aim to deliver a compact device in a package that can be used without needing any engineer as explained in the “Future work” section. This device will be unique in that it recognises the warning signs of a pending emptying of the bladder via tracking expansion of the bladder volume over time, and will wake the patient up in time to prevent it. This process is customised or tuned to an individual patient’s bladder volume trigger point. This more accurate advanced warning system will help the children to alter their behaviours over time, reducing the frequency of nocturnal enuresis [10, 11] through learning bladder control over time. In this manner, our further development of the device is aimed to adjust the triggering point further for larger volumes of urine as the child learns the time for voiding need and consequently learn the control of his/her nerves. The phases of the application are explored in a scenario for 8 years old boy with a normal BMI in Figs 14 and 15.

There have been several studies to find a solution for NE sufferers [10, 11], however, no product that can predict a pre-void occurrence in the market so far. In [10], the authors concluded that the results were not satisfactory due to the difficulty in transducer orientation and in detecting proper signals for different morphology types, sexes and age groups using a pre-set threshold value. We could not find any software system that learns and evaluates the changing characteristics intelligently and triggers an alarm accordingly in this study either. This study shows that a static system can not be successful in determining pre-void need within the complex dynamic environment of the bladder with respect to morphology types, sexes, age groups and even specific individual features. In [11], a bladder volume alarming was tested on children to find out the effect of similar devices on children to cure NE rather than developing a compact device. The device used in the study is a portable containing an onboard computer that provides a hard copy printout of the scanned images and the bladder volume based on image analysis different from our techniques. The components in this study were not ergonomic and comfortable for the children to use. In addition, no intelligent software was used to evaluate the changing characteristics of the bladder with respect to various morphology types, sexes and age groups. For the success of the similar devices, correct components with proper properties should be forged to create a successful device. Most importantly, the ergonomic design and ease-of-use with wireless technologies are the other key factors for a device to be successful. A robustly working device may not be used if it is not comfortable enough. Development of such a mechatronic device requires collaboration of the different disciplines (mechanics, electronics, information technology, design, medical field) to create a synergy. We believe that we created this collaboration to realise the objectives in the project.

The results to date have confirmed that a targeted intelligent device is feasible and would meet a significant clinical need. If children and families are to be motivated to use alarm system to improve NE the system needs to be accessible, affordable and effective at minimising wetting by inhibiting the event of a wet bed, by early alerting. The system needs to be easy to use with data intuitively informing an individual management approach, working on optimising design, wearability and userfriendliness. The main advantage of our techniques using a single-element transducers is its simplicity, safety and low cost, and is especially suitable for miniaturisation and thus monitoring purposes and most importantly private use.

The *Se* and *Sp* values of 1/2 triggering point that is not the imminent voiding point are 0.98 and 0.96 respectively, which are quite high. However, 3/4 is the imminent voiding point and calculations should be done with respect to this point as the sleep interruption at 1/2 triggering point is not that necessary. The *Se* and *Sp* values of 3/4 triggering point are 0.89 and 0.93 respectively. The *Se* value of 0.89 indicates that 11 alarm out of 100 might be false alarm causing sleep interruption with no reason. The *Sp* value of 0.93 indicates that in 7 out of 100 times no alarm sounds when the child should have been woken up, resulting in a wet bed. Although the number of false decisions is not that large with respect to 3/4 triggering point, these success rates should be improved in order not to cause any bed-wetting or unnecessary sleep interruption and could be improved as mentioned in the following paragraphs and in the “Future work” section in detail and we are developing an autonomous device accordingly for the time being under the My-PAD project.

In this study, a transducer with a static matching layer was employed, which creates a big reflection at the abdomen (Figs. 1 and 2). Now, a US company is developing a transducer with a tapered matching layer that suits the soft tissue best as explained in the Supplementary materials (Fig. 1 in the Supplementary materials). With this transducer emitted pulses will be able to be transmitted through the abdomen and more sensitive echoed pulses will be able to be acquired. We aim to use three of these sensors with different angles (Fig. 17a) to face the bladder in the future development of the device to both ensure that the acquired signal is related to the bladder and to increase the *Se* and *Sp* values via a plurality of transducers interchangeably.

**Fig. 16:**
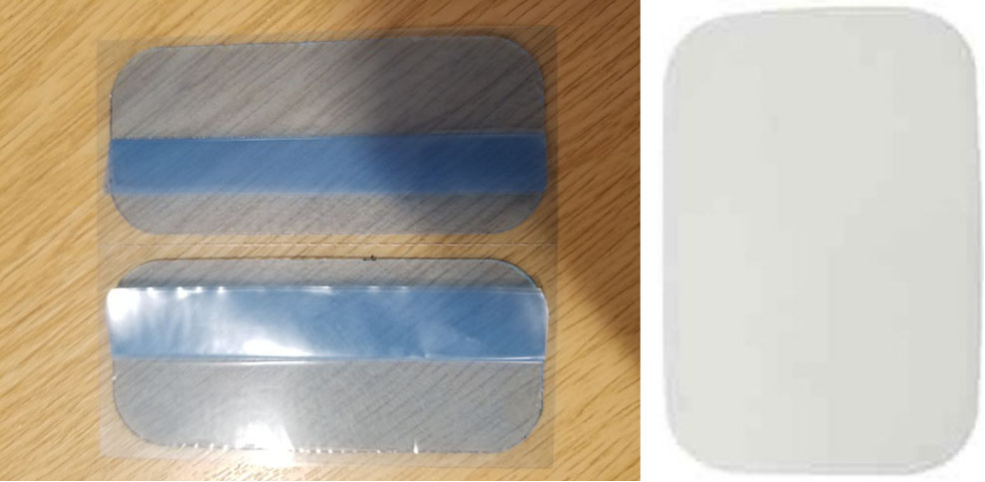
Self-adhesive gel pads: both sides to stick the hypo-gastric region at the proximal side and probes at the distal side

**Fig. 17:**
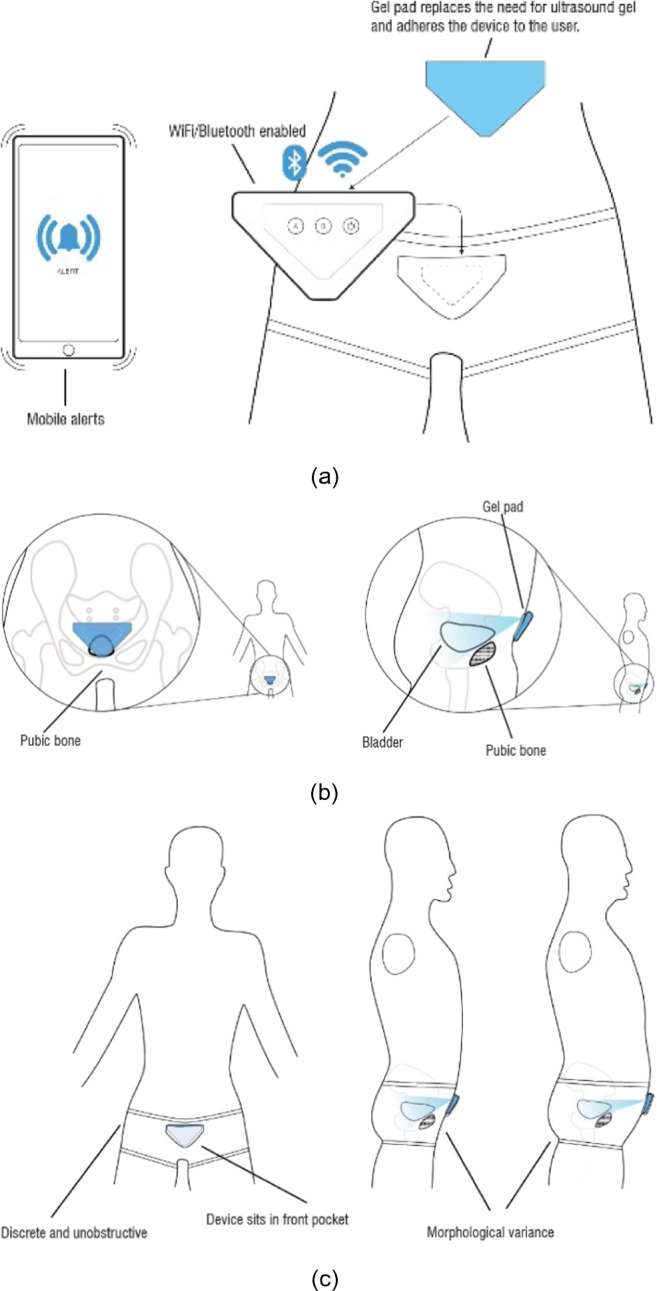
Design concept for a wearable support garment: A performance critical aspect of this technology is its adoption by the end-user across various psychophysiological requirements. This approach provides this in two critical areas, more specifically: i. Ease of application/use by a non-technical person to the body, by user or carer offering instant accurate location of device. ii. Offers a discrete undergarment opportunity that can be designed so as not to convey a medical condition in everyday life events. i.e. school environments. **a** General framework of the concept; **b** Positioning with respect to bladder; **c** Representation with respect to morphologic types: i. the devices shape in relationship to the pubic region, this will be graded to fit population types. ii. the garment will provide tension to the rear of the device maintaining contact with the skin. iii. the inner pocket of the proposed garment has a window which enables the self-adhesive gel pad which is adhered to the body side of the device to protrude through and adhere to the skin. Various fit ranges can be developed to accommodate population morphological types

An acoustic US gel was used between the transducer and skin to transmit the pulses into the human body. This is not comfortable for the children, and it is not easy to keep the probes to stick to the abdomen for self use to remove the air between these two interfaces, which causes an acoustic coupling risk. This acoustic coupling risk has been mitigated by a dual approach of identifying and testing self-adhesive skin-interfacing gel pads which are used for the prolonged attachment of heart rate monitor devices. We aim to use similar pads as shown in Fig. 16 through a new design for a washable undergarment. To address the comfort concerns a team at the University of Central Lancashire specialised in industrial design and human factors research methods, and user informed design solutions, more specifically in the fields of medical products has been involved. These have produced a design concept for a wearable support garment to keep the device in firm contact with the body, and in an optimum position with appropriate angles for obtaining bladder measurements for both male and females and for different population morphology types. This is illustrated in Fig. 17 by which three main aspects maintaining its position are explained whilst enabling comfort. The combination of the devise proportions and geometry, support garment and the gel pad will ensure the device is firmly located. The use of plurality of transducers interchangeably will be more effective using this appropriate undergarment.

We will be collaborating with Royal Preston Hospital Radiology Department to acquire the different levels of volume in the bladders of our volunteers using hand-held US scan devices. As such, we will be able to compare the results of our technique to the results of a conventional US device.

Volunteer and phantom measurements showed that bladder volume assessment on the basis of nonlinear wave propagation is feasible [21] as explained in the Supplementary materials (Figs. 8 and 9). The relationship between the detected harmonic components and urine volume in the bladder will provide an important ability to our study to determine the required threshold volume to trigger an alarm. This property will be incorporated into our US device to result in better *Se* and *Sp* values.

In the long run, large collected data samples from different sexes, age groups, morphology types can be processed to train a stand-alone system that can be employed for larger range of NE patients without training phase. Beyond this study, there are numerous other areas of application i.e. elderly care (geriatric) settings, stroke patients [32], diagnosis of urinary retention and veterinary science in which My-PAD can be of potential benefit. The approach being taken with this study is to develop an external alarm that will wake the child up when a possible urinary incontinence event is predicted rather than merely responding to the presence of moisture after an event has taken place as conducted by the current alarm devices in the market. More particularly the present study relates to methods and apparatuses for treating urinary incontinence, suitably by providing pre-void alerts that allow a patient to void in a dignified manner.

## Conclusion

The feasibility study shows that the development of a pre-void alarm device to treat NE is viable by combining several techniques and expertise. In this manner a customisable device funded by National Institute for Health Research (NIHR) is being developed for patients suffering from NE based on the findings in the study.

The potential benefit of this study are as follows: (i) The device to be built is aimed to find an alternative to currently available wet alarms. It can also help with initial consultations to characterise the bladder expansion patterns; (ii) The children with NE will adapt to the safe, non-invasive, customisable and proactive device to void in a dignified way avoiding the trauma and stigma of bed-wetting, which will enhance quality of their life, and consequently their psychology positively with dry nights; (iii) The device will help the children reach stable dryness through learning bladder control over time; (iv) Young people who could not be cured with the current approaches and many other people who never seek help because they are too embarrassed will be eager to use the device; (v) The cost of using the device will be lower than other long term approaches either for families and NHS (The National Health Service); (vi) The number of hospital visits of children with NE and their families using the My-PAD device will be reduced significantly, and consequently health resources can be reallocated; (vii) With minor modifications, the device can be implemented more widely and optimised for example in use with stroke patients and elderly people who are vulnerable to urinary incontinence and patients with neuropathic bladder; (viii) The smartphone application using the ML techniques in order to provide customisable settings specific to the characteristics of the sufferers will make the device more successful to determine the bladder filling level and to trigger the desired pre-void alarm accordingly by taking the changing behaviours of the users into consideration.

## Future work

Patient and public involvement (PPI) is critical to improve the proposed medical device and to ensure the user acceptance of the device. The geometry, wearability, usability, warning method and optimisation of the device is being iterated through the PPI groups established by our collaborates Preston Royal Hospital and our partner charity ERIC (Education and Resources for improving Childhood Continence).

Our design group at University of Central Lancashire has worked on a design concept for a wearable support garment along with a gel-containing comfortable absorbent pad for the interface between the transducer and skin to keep US interfacing component of the device in firm contact with the body, and in an optimum position for obtaining bladder measurements. The garment can be separated from the device to be washed. An ergonomic wireless US device that has the similar features mentioned throughout this manuscript is being developed by our collaborator in Industry, Leoceur Electronique that has safely applied their US technology in medical applications (dentistry and brain surgery) in trials and gives us access to a team of experts in ultrasonic technology. Integration of all required components in an appropriate device case is being carried out by the University of Central Lancashire. Other sensors such as temperature, moisture detectors and movement measurement (i.e., angular accelerometer) for determining postural changes are being incorporated into the device to enhance warning performance and self-customising features.

## Electronic supplementary material

Below is the link to the electronic supplementary material.

(PDF 1.83 MB)

## Footnotes

1 The first gate is specified for the anterior wall and the second gate is specified for the posterior wall of the bladder.

2 BMI is measured using the formula built by the University of Oxford, BMI = 1.3 ∗ *w**e**i**g**h**t*(*k**g*)/*h**e**i**g**h**t*(*m*)^2.5^ = 5734 ∗ *w**e**i**g**h**t*(*l**b*)/*h**e**i**g**h**t* (*i**n*)^2.5^ (https://people.maths.ox.ac.uk/trefethen/bmi.html).

3 TP: “voiding need” is correctly identified as “trigger alarm”; FP: “no voiding need” is incorrectly identified as “trigger alarm”; TN: “no voiding need” is correctly identified as “no alarm”; FN: “voiding need” incorrectly identified as “no alarm”.
