# Supplementary material for: Feasibility study of intelligent autonomous determination of the bladder voiding need to treat bedwetting using ultrasound and smartphone ML techniques: Intelligent autonomous treatment of bedwetting
Source: Med Biol Eng Comput. 2018 Dec 26;57(5):1079–97. doi: 10.1007/s11517-018-1942-9 (PMC6477014; doi:10.1007/s11517-018-1942-9)
Supplement: Supplementary file 1 — (PDF 1.83 MB) [file 11517_2018_1942_MOESM1_ESM.pdf]

Noname manuscript No.
(will be inserted by the editor)

Feasibility study of intelligent autonomous determination of the bladder voiding need to treat bedwetting using ultrasound and smartphone ML techniques - Supplementary materials -

Received: date / Accepted: date

1 Basics of ultrasound physics with respect to the employed techniques in the study

The wavelength and frequency of US are inversely related, i.e., US of high frequency has a short wavelength and vice versa [1]. The propagation/depth of US in body with respect to the frequency is depicted in Table 1. High US frequencies are accompanied by high attenuation losses because of the short wavelength. In other words, higher frequency sound waves have a larger attenuation coefficient and thus are more readily absorbed in tissue, limiting the depth of penetration of the sound wave into the body; the lower the frequency, the less the attenuation and the higher the propagation/penetration. Higher frequencies are usually applied to get higher resolution images close to the skin. No imaging modality is used in our approaches. Therefore, we employed single element transducers as a transmitter and receiver simultaneously with a low frequency of 2.2 MHz to allow a longer range of wavelength to go a longer distance as shown in Table 1 (notice the distance, 20 cm for 2 MHz). The wavelength for the soft tissue in our case: $\lambda = 1540/2.2 * 10^6 = 0.7$ mm where 1540 is the mean velocity of the tissue.

Sound energy is attenuated or weakened as it passes through the tissue because parts of it are reflected, scattered, absorbed, refracted or diffracted. What we are interested in most is reflection or echoed pulses in this study. When a sound wave travels through a tissue with a uniform density and encounters an interface with a different density, some of that sound wave bounces back as an echo [2]. Reflection occurs between two adjacent tissues/media and the amount of reflection is mainly related to the acoustic impedance difference of these two tissues/media; the greater the acoustic impedance difference, the greater the amount of reflection. The acoustic impedance (Z) is denoted by Rayl ($kg/(s.m^2)$) and is defined as $Z = p.c$ where p is the density (kg/m^3) of the media, c is the velocity of the media. The acoustic impedances of related tissues/media and reflection of US energy at several interfaces are presented in Tables 2 and 3 [3] respectively. The reflection at the interface of two media is calculated using the formula, R = (Z1-Z2/Z1+Z2) [4] where Z1 represents the impedance value of the proximal side of the interface and Z2 represent the impedance value of the distal side. No refraction occurs at the interface if the beam is perpendicular no matter what the sound speed difference is between the two materials. On the other hand, during the application of US non-perpendicularly, the refraction increases when the difference between the sound velocities in two media increases. Soft tissues are mostly (60%) composed of water and values for average velocity are similar, varying only about 10% [5]. Therefore, there are no such large difference throughout the media in the ROI of bladder as shown in Table 4 [4]. The refraction between soft tissue (c= 1540) and the fluid/urine (c= 1551) is 1.2° for a beam degree angle of $sinQ_i = 28.8$ using the formula, Snell's law, $sinQ_t/sinQ_i = c_2/c_1$

Table 1: Depth of US penetration in the body with respect to frequency.

Frequency (MHz)	1	2	3	5	10	20
Depth of Penetration (cm)	40	20	13	8	4	2

Financial support for this study was provided entirely by a grant from the National Institute for Health Research (Invention for Innovation, i4i; MyPad - Intelligent Bladder Pre-void Alerting System; II-LA-1116-20007). The funding agreement ensured the authors' independence in designing the study, interpreting the data, writing, and publishing the report.

Address(es) of author(s) should be given

Table 2: Acoustic impedance of several media at 37 C° $(kg/s.m^2 * 10^6)$.

Substance	Bladder	Water	Urine	Air	Bone	Muscle	Fat	Soft tissue	Kidney	Blood	Liver	Lung
Imp	1.64	1.48	≈ water	0.0004	7.8	1.71	1.34	1.63	1.63	1.65	1.65	0.18

Table 3: Reflection of US energy at several interfaces.

Interface	Muscle/fat	Muscle/air	Bone/muscle	Bone/fat	Soft tissue/air	Soft tissue/water	Anterior wall/air	Anterior wall/urine
Reflection (%)	1	≈ 99.9	41	49	99.9	0.2	≈ 99.9	5

Table 4: Refraction of US at several interfaces.

Interface	Transmitted angle	Refraction
Bone/soft tissue	10.9	19.1
Muscle/fat	27.1	2.9
Muscle/fluid	28.8	1.2
Muscle/blood	29.2	0.8

since the velocities in both media are almost same [4], which makes easier to acquire echoed pulses generated from the consecutive interfaces.

The Z value of the window of the transducer should be almost the same Z value of the soft tissue, namely abdomen for the better transmission of US beams emitted from the transducer. One of the ways is to use the quarter-wave matching to determine the impedance value of matching layer with regard to the piezoelectric element to minimize the reflection right in front of the transducer surface using the single quarter-wave matching formula, $Z_{match} = \sqrt{Z_t * Z_{st}}$ where Z_t is the impedance value of the transducer and Z_{st} is the impedance value of the soft tissue. For instance, a piezoelectric slab has a Z value of 30 x 10^6 and a soft tissue (e.g., abdomen) has a Z value of 1.63 x 10^6. In this case, the matching layer on the transducer should have a Z value of 7.10^6 to minimize the mismatch as calculated: $Z_{match} = \sqrt{Z_t * Z_{st})} = \sqrt{(30x10^6 * 1.63x10^6)} = 7.10^6 rayls$, which provides efficient transmission of sound waves from the transducer element to soft tissue and vice versa by reducing the reflections at the interface of transducer/abdomen. Proper matching $(Z1 - Z2 \approx 0)$ is preferable for the transmission of US wave through the deep abdomen with very little loss without much reflection. Alternatively, in a more effective way, a tapered matching layer in which a number of layers of materials (i.e., multiple matching layers) taper the impedance from that of the crystal to that of tissue to transmit

Table 5: Half value thickness (HVT) is the distance which reduces the intensity of an US signal to one half of its original propagated signal in terms of the frequency and the properties of the tissue/media it propagates.

HVT (cm)	Water	Urine	Muscle	Fat	air	Bone
2MHz	340	≈ water	0.75	2.1	0.06	0.1
5MHz	54	≈ water	0.3	0.86	0.01	0.04

100% of the energy into human body as illustrated in Fig. 1 can be utilized. Time Gain Control function is employed to adjust the amplitudes as a function of time by increasing the receiver gain with respect to attenuation as illustrated in 2.

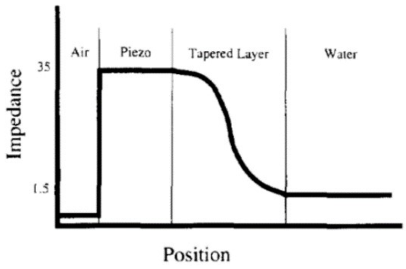

Fig. 1: Plot of impedance vs. position for a piezoelectric transducer with a tapered matching layer [6].

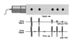

Fig. 2: Time Gain Control (TGC): The top signal corresponds to echoes from equal reflectors, where attenuations decreases the amplitude as the distance to the reflector increases. TGC compensates for attenuation in tissue by increasing the receiver gain as a function of time [4].

The intensity of US pulse reduces as it penetrates through the tissues/media regarding the frequency. Half value thickness of related tissues/media with respect to two frequencies, namely 2 and 5 MHz are shown in Table 5 [3]. Therefore, amplitude of the echoed pulses decreases with increasing depth and Distance Amplitude Correction (DAC) (i.e., Time Gain Compensation) is employed to amplify the echoed pulses with respect to distance propagated. Notice the very small acoustic loss in urine in Table 6 [7]. Total attenuation is calculated using the formula, Total attenuation (dB) $= \alpha(dB/cm) * d(cm)$ where α is attenuation coefficient and d is the distance.

The relationship of US beams and the bladder in broader perspective is illustrated in Fig. 3. This re-

Table 6: Properties of several tissues.

Tissue	Sound velocity (m/s)	Density (g/cm^3)	B/A	Acoust.loss (α) ($dB/(mm.MHz^b)$)	b
Fat	1436	0.928	9.6	0.30	0.9
Muscle	1550	1.060	5.8	0.05	1.1
Blood	1584	1.060	6.0	0.01	1.2
Urine	1551	1.025	6.1	0.00047	1.67
Water	1524	0.993	5.4	0.00014	2.0

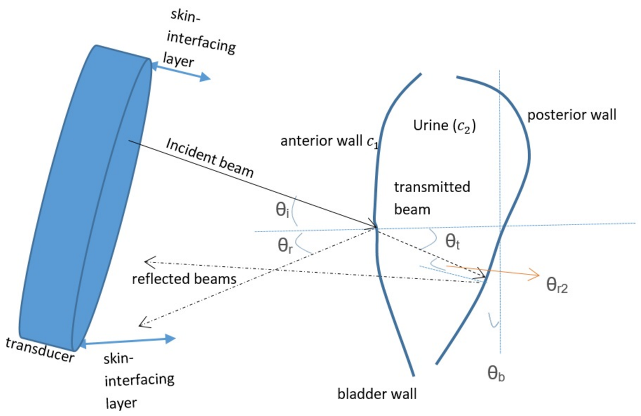

Fig. 3: Interaction of US beams and the bladder.

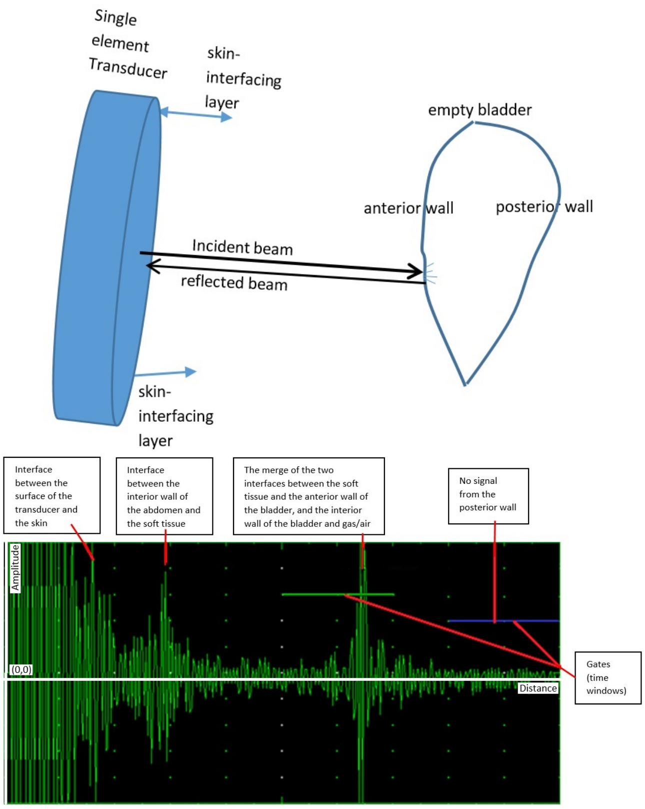

Fig. 4: Interaction of US beams and the bladder with respect to an empty bladder: a single-element US transducer and respective propagation and attenuation signals; the pulsed signal is almost completely reflected (99%) and the remaining propagated signal after the anterior wall of the bladder is scattered in all directions in a non-uniform manner due to emptiness within the bladder.

lationship is depicted with respect to empty and full bladder in Figs. 4 and 5 respectively. The reflection of the pulsed signal from the anterior wall of the bladder where the bladder is empty is 99.9%, which illustrates the difficulty in transmitting emitted pulses beyond the distal side of the bladder. It is 5% where there is urine by which a pulsed signal can reach the posterior wall without any difficulty. Measuring the movement of the front and back wall of the bladder is a strong indication of the level of expansion and the amount of urine inside the bladder. An output example of a phantom filled with urine is given in Fig. 6 to illustrate and understand this feature of the US physics.

The nonlinearity of the medium through which a finite-amplitude ultrasonic wave propagates described in the Goldberg factor [8] which is denoted as

$$G = 2\pi f p(1 + 0.5\beta)/(pc^3\alpha) \quad (1)$$

in which β is the coefficient of nonlinearity which the generation of higher harmonics depends on it. Acoustic medium parameters such as the acoustic non-linearity parameter β are known to be temperature dependent.

[9] indicates the ability of the media to form higher harmonics to an originally pulse wave of finite amplitude during its propagation away from the wave source; it is also dependent on frequency f, and the excitation pressure p. When G is less than unit, the attenuation dominates the non-linearity, and for any G value above 1 the non-linearity takes the lead-according to effectiveness [10]; fixing the stimulation frequency and the pressure at 3MHz and 1MPa, the G factor gets the value of 104 for urine, and 0.27 for soft tissues of body as shown in Fig. 7 [7]. This demonstrates more intense nonlinearity behaviour for urine (liquid) than other soft tissues (solid). Within soft tissues, non-linear processes also take place, but are attenuated and modified as a result of different acoustic characteristics, like high acoustic absorption. The use of harmonics in medicine interaction with contrast agents (e.g., in the veins) creates higher frequency harmonics (e.g., > 2 times the transmitted frequency) to obtain high resolution of imaging. Second harmonic is usually being used for tissues because amplitudes of the subsequent harmonics are decreasing due to significant attenuation on tissues regarding very high generated frequencies. The transmit frequency of the transducer should be set to half of the centre frequency of the transducer to be able to

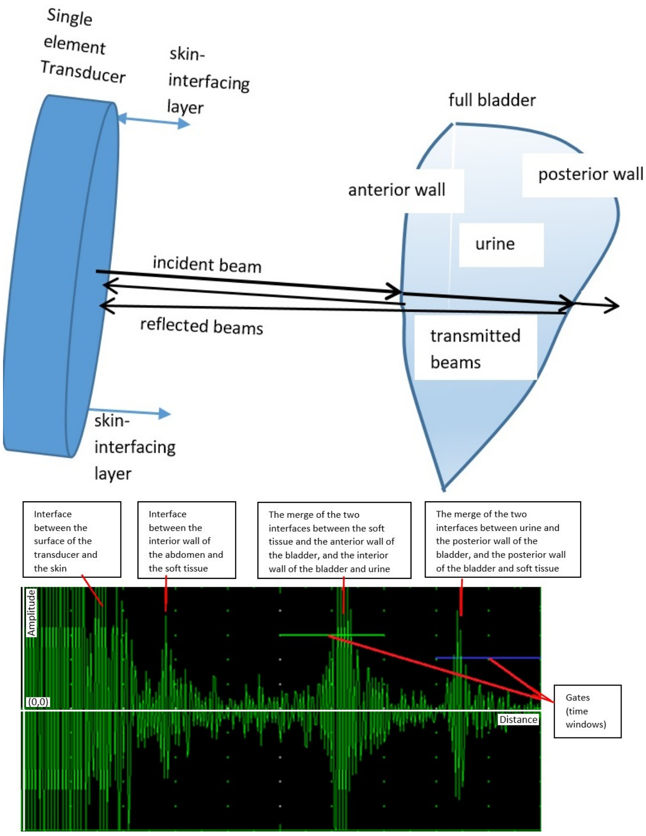

Fig. 5: Interaction of US beams and the bladder with respect to a full bladder: single-element US transducer and respective propagation and attenuation signals where the reflection is 5% and propagation signals after the anterior wall of the bladder do not lose their strength because the urine inside the bladder causes little attenuation

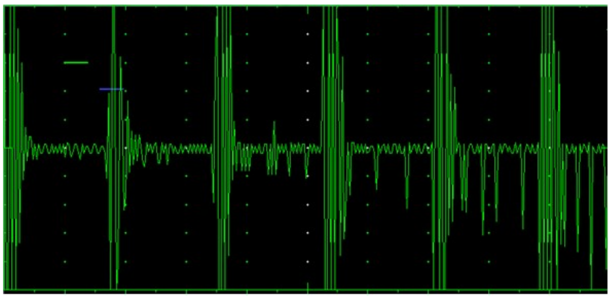

Fig. 6: Phantom test with full of urine and air after the posterior wall: the signals reflect back and forth with a very little acoustic loss regarding urine (0.00014 for urine) and a very big reflection (95%) just like a ping-pong ball between anterior and posterior wall.

receive with the same transducer at the second harmonic frequency. We already have a natural contrast agent such as urine (Fig. 7); blood in the veins needs

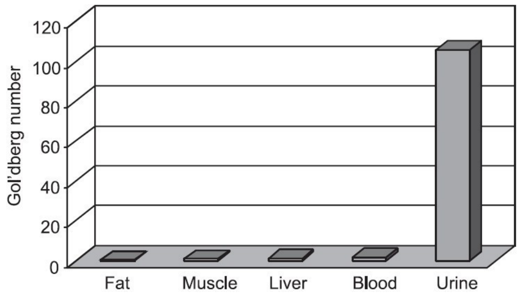

Fig. 7: Goldberg number calculated for different media at a transmit frequency of 3 MHz and acoustic pressure of 1MPa for creating harmonics based on nonlinear wave propagation.

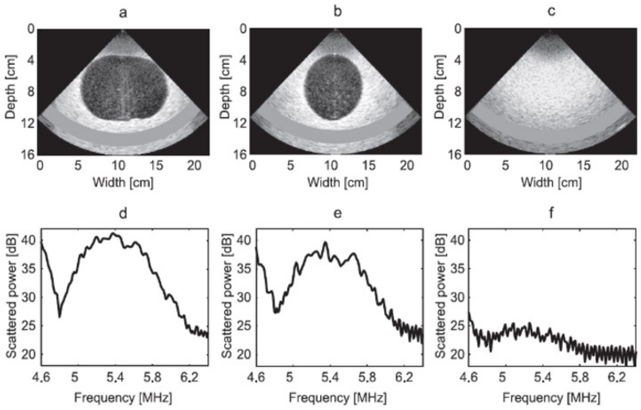

Fig. 8: Correlation of harmonic components (second harmonic) to the amount of liquid. The second harmonics with respect to the urine volume in the bladder. a) full phantom, b) half phantom c)empty phantom, d) acquired scattered power in dB from image in (a), e) the image in (b), and f) the image in (c).

contrast agents where Goldberg number is too small when compared to that of urine that does not need contrast agents to generate harmonics. Therefore, using only a single element transducer that generates a diverging beam, the information about the amount of liquid present in the irregular 3D space of bladder is instantaneously translated, through the physics of US, nonlinear wave propagation, to the amount of urine within echoes as depicted in Fig. 8 from a phantom and Fig. 9 from a volunteer [7], which can measure the volume of the bladder based on different and complex shapes of bladder at different filling stages.

2 Safe use of ultrasound in medicine

Most of the standards, guidelines and regulations put forth by major US organizations about the safe use of

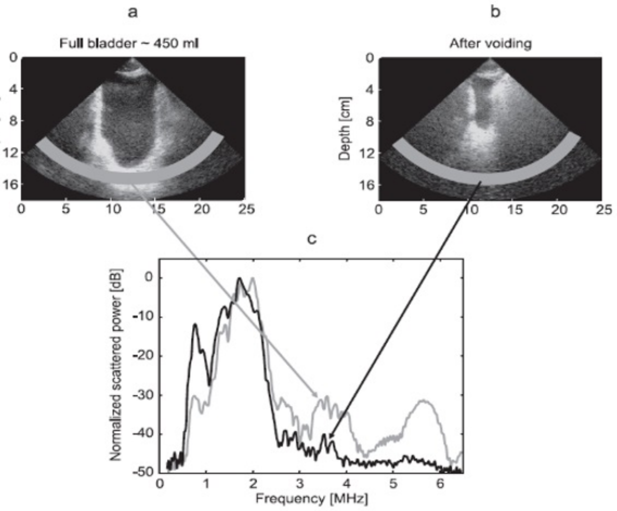

Fig. 9: Volunteer measurement: (a) full bladder, (b) bladder with less urine, (c) corresponding harmonic measurement in dB.

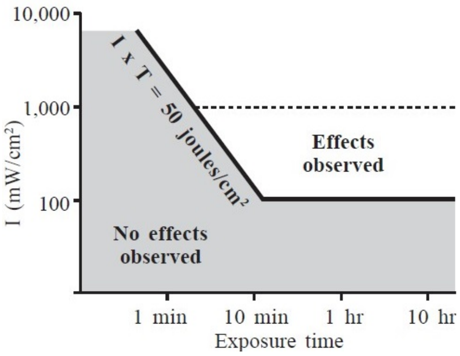

Fig. 10: Intensity (spatial peak-temporal average) versus time curve relevant to the production of bioeffects in mammalian tissue [4].

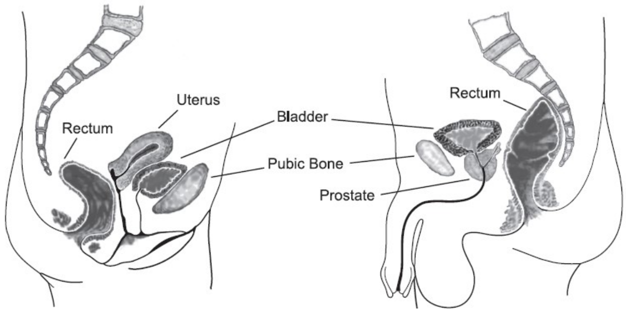

Fig. 11: Median section of the pelvis of an adult female (left) and adult male (right) [7].

US had been specified before 2000 and these standards are still being used. These recommendations and guidelines offer valuable information to help users apply diagnostic US in a safe and effective manner with regards to diagnostic modalities (e.g., A-Mode, B-Mode, Doppler, harmonic imaging using contrast agents).

The two biophysical indices given by the ODS (output display standard) are Thermal (measured as the thermal index, TI is the ratio of acoustical power produced by the transducer to the power required to raise the temperature of the tissue by 1 C° [11])) and non-thermal (measured as the mechanical index, MI value is computed from the peak rarefactional pressure and the frequency, and is intended to estimate the potential for mechanical bioeffects). The higher the index value, the higher the probability for bioeffects occurring; values of less than 1 are generally considered safe [11]) [11]. The TI provides information about tissue temperature increase, and the MI provides information about the potential for cavitation [7]. As a sound beam passes through tissue, it undergoes attenuation. A significant fraction of this attenuation is due to absorption which causes the heat. For low power US, the heat deposited is quickly dissipated whereas some concern is warranted with pulsed Doppler and colour flow imaging equipment, where high power levels and time average intensities may give large TI values [12]. The principal non-thermal (mechanical) interactions deal with the generation, growth, vibration and possible collapse of microbubbles within tissue; this behaviour is known as cavitation; two types of cavitation exist: stable cavitation refers to the creation of bubbles that oscillate with sound beams; transient cavitation refers to the process in which the oscillation grows so strong that the bubbles collapse violently, producing very intense, localized effects [11]. In situations where the TI or MI is greater than 1, the exposure time in any one location should be kept as short as possible [12].

Fig. 10 shows the intensity (I) versus time (T) curve regarding the production of bioeffects in mammalian tissue according to the experimental results performed by The American Institute of Ultrasound in Medicine (AIUM) in 1992. The line (below 100 mW/cm2) divides the data into a region of positive biologic effects and a no-effect-observed region. The line follows a curve for intensity, greater than 100 mW/cm^2 in which I x T = 50 J/cm^2. Below 100 mW/cm^2 findings by using large beams from unfocused transducers indicate that none of the effects for which the researches were looking could be produced, no matter how long the exposure is [4]. No bioeffects are observed for the intensity levels below 100 mW/cm^2 even though the exposure time is increased up to 10 hours. Again, there are no bioeffects with 1000 mW/cm^2 less than 5 min exposure time and with 100000 mW/cm^2 for applications of US in several seconds. For focused transducers, greater

time average intensities are tolerated because any heat deposited over a small focal area is dissipated easily to the surrounding unexposed tissue [4]. Taking this into considering additional experimental results in addition to the earlier results in 1992, AIUM extended the observation, claiming that intensities as 1 W/cm2 (dotted line in Fig. 10) have led to no observed effect in mammalian tissues exposed to highly focused sound beams [4]. Fig. 10 indicates that diagnostic US pulses, at least those at high levels and applied for fairly long exposure time, can cause a bioeffect [4]. The longer pulses and higher pulse-repetition rates typically used in pulsed Doppler exposures result in higher average intensities compared to B-mode imaging or A-mode presentation, and therefore involve a greater risk of producing biological effects, particularly from US-induced heating [13].

AIUM evaluated epidemiologic studies and concluded that: 'Widespread clinical use over 25 years has not established any adverse effect arising from exposure to diagnostic US' [14,15].

3 Expansion of the bladder

The urinary bladder is a muscular organ in the pelvis, just above and behind the pubic bone [7,16]. Urine is made in the kidneys [16] and transported from the kidneys to the bladder via two ureters, which are connected at the back of the bladder; the ureters contain valves proximal to the bladder to prevent the urine from flowing back to the kidneys; finally, the urine is transported outside the body via the urethra [7]. Because urine passes through the penis, the urethra is longer in men (8 inches) than in women (1.5 inches) [16]. The complete system of kidneys, ureters, bladder and urethra is called the urinary system, a part of which is delineated in Fig. 11.

The following echoed pulses were observed from a seven year old volunteer: x-axis corresponds to distance (ROI is 20 cm) and y-axis corresponds to amplitude of the echoed pulses (only positive values are taken into consideration).

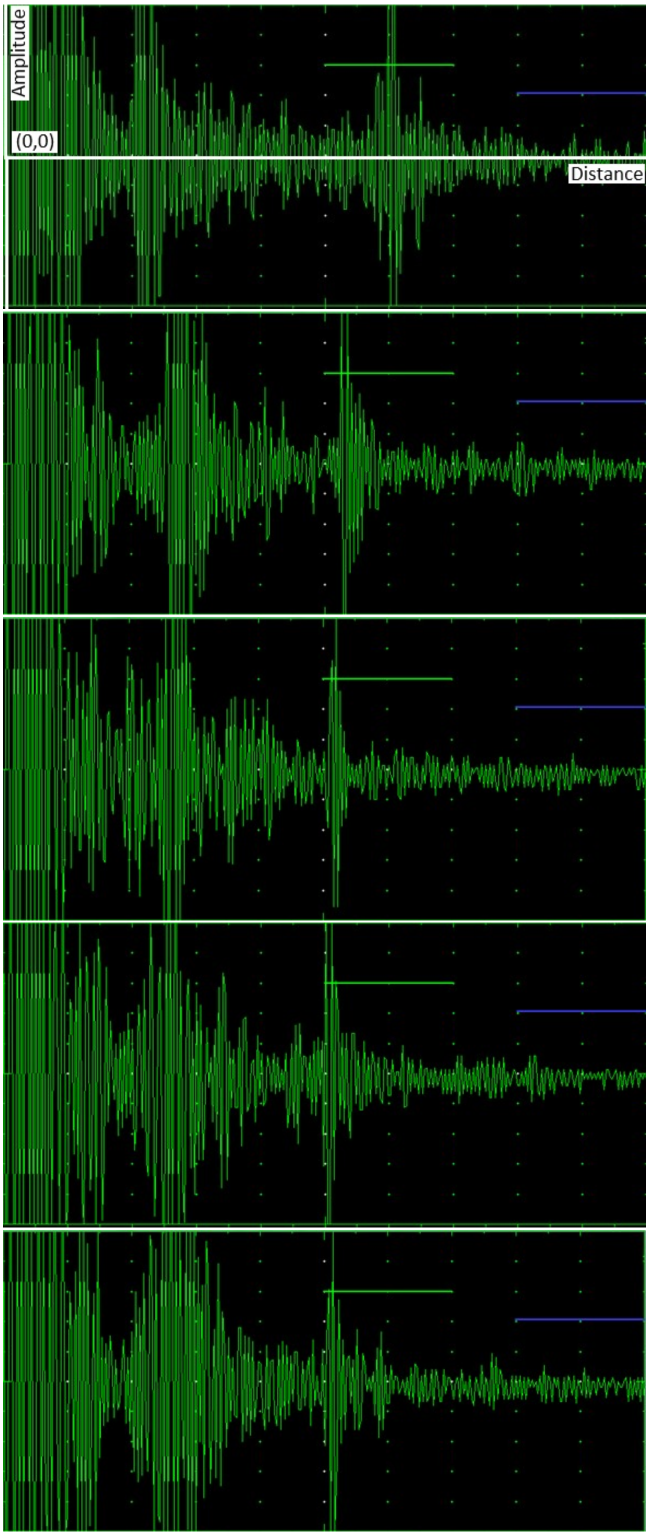

Fig. 12: Empty bladder: no attenuation signal from the posterior wall (second purple gate) and an attenuation signal from the anterior wall (first green gate). These signals were obtained in 20 minutes intervals.

References

1. V. Chan and A. Perlas, "Basics of ultrasound imaging," in *Atlas of Ultrasound-Guided Procedures in Interventional Pain Management*, S. N. Narouze, Ed. Springer, 2011.
2. D. Rigby and F. Housami, "Using bladder ultrasound to detect urinary retention in patients," *Journal of Urology*, vol. 105, no. 21, 2009.

3. N. Tole and H. Ostensen, "Basic physics of ultrasonographic imaging)," 2005. [Online]. Available: http://apps.who.int/iris/bitstream/10665/43179/1/9241592990_eng.pdf
4. J. A. Zagzebski, *Essentials of ultrasound physics.* St. Louis : Mosby, 1996, includes bibliographical references and index.
5. J. Bronzino, *Diagnostic ultrasound imaging.* California, USA: Elsevier., 2004.
6. M. I. Haller and B. T. Khuri-Yakub, "Tapered acoustic matching layer." Proc. IEEE. Ultrason. Symp, 2009, pp. 505–508.
7. E. J. W. Merks, "Instantaneous ultrasonic assessment of urinary bladder volume," Ph.D. dissertation, Erasmus University Rotterdam, Optima Grafische Communicatie, Rotterdam, the Netherlands, 6 2009.
8. L. Bjrn, "Introduction to nonlinear acoustics," *Physics Procedia*, vol. 3, no. 1, pp. 5 – 16, 2010, international Congress on Ultrasonics, Santiago de Chile, January 2009. [Online]. Available: http://www.sciencedirect.com/science/article/pii/S1875389210000040
9. K. W. A. van Dongen and M. D. Verweij, "A feasibility study for non-invasive thermometry using non-linear ultrasound," *International Journal of Hyperthermia*, vol. 27, no. 6, pp. 612–624, 2011. [Online]. Available: https://doi.org/10.3109/02656736.2011.599357
10. M.-X. Tang, J. Loughran, E. Stride, D. Zhang, and R. J. Eckersley, "Effect of bubble shell nonlinearity on ultrasound nonlinear propagation through microbubble populations," *The Journal of the Acoustical Society of America*, vol. 129, no. 3, pp. EL76–EL82, 2011. [Online]. Available: https://doi.org/10.1121/1.3544677
11. K. H. Ng, "International guidelines and regulations for the safe use of diagnostic ultrasound in medicine," *Journal of Medical Ultrasound*, vol. 10, no. 1, pp. 5 – 9, 2002. [Online]. Available: http://www.sciencedirect.com/science/article/pii/S0929644109600175
12. B. B. Stanley, G. R. T. Haar, M. C. Ziskin, and H.-D. Rott, "International recommendations and guidelines for the safe use of diagnostic ultrasound in medicine," *Ultrasound in Medicine and Biology*, vol. 26, no. 3, pp. 355 – 366, 2000. [Online]. Available: http://dx.doi.org/10.1016/S0301-5629(00)00204-0
13. W. D. O'Brien and D. S. Ellis, "Evaluation of the unscanned soft-tissue thermal index," *IEEE Transactions on Ultrasonics, Ferroelectrics, and Frequency Control*, vol. 46, no. 6, pp. 1459–1476, Nov 1999.
14. M. D. Laurel and V. Rosslyn, "Aium: Standard for real-time display of thermal and mechanical indices on diagnostic ultrasound equipment," 1993.
15. M. D. Laurel, "Aium: Bioeffects and safety of diagnostic ultrasound," 1993.
16. MedicineNet, "Medical anatomy and illustrations," http://www.medicinenet.com/image-collection/bladder_picture/picture.htm, 2009, accessed: 2015-05-04. [Online]. Available: http://www.medicinenet.com/image-collection/bladder_picture/picture.htm

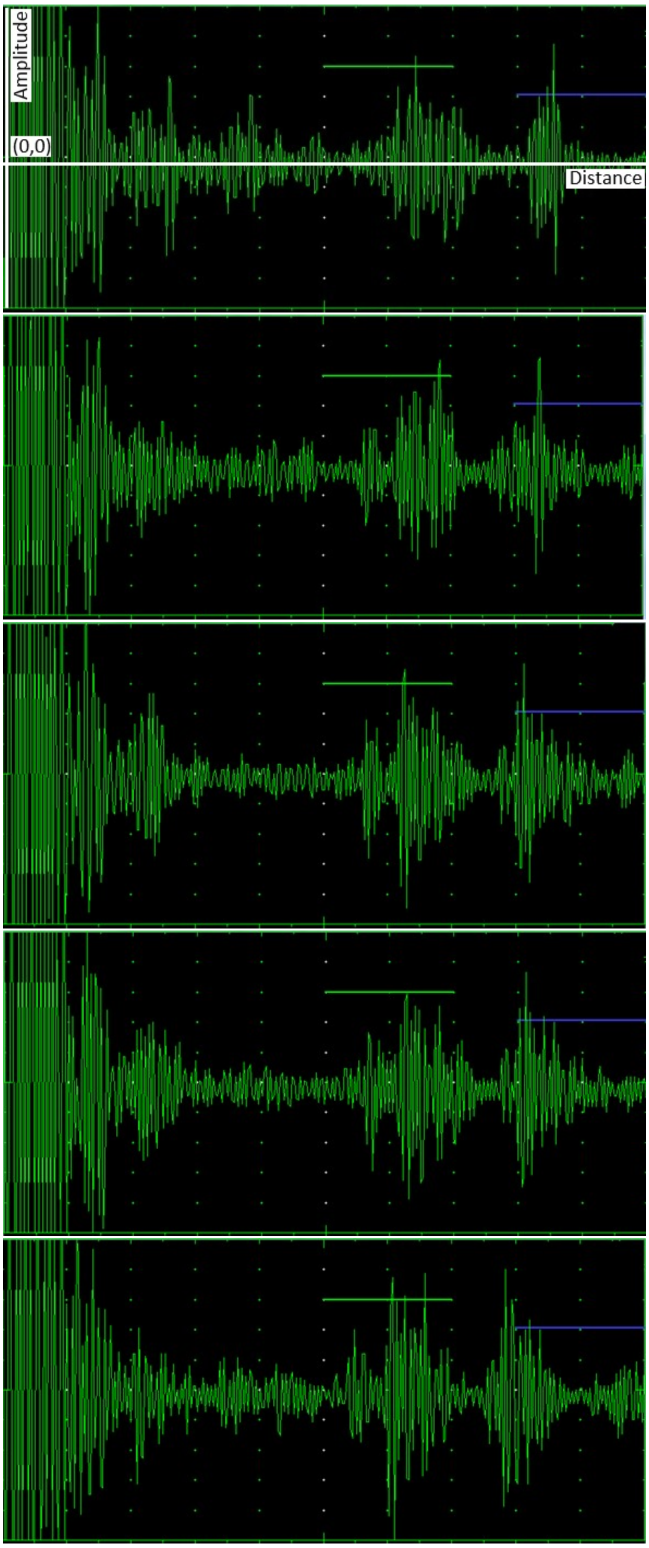

Fig. 13: Half bladder expanding: an attenuation signal from the posterior wall (second purple gate) and an attenuation signal from the anterior wall (first green gate). These signals were obtained in 15 minutes intervals.

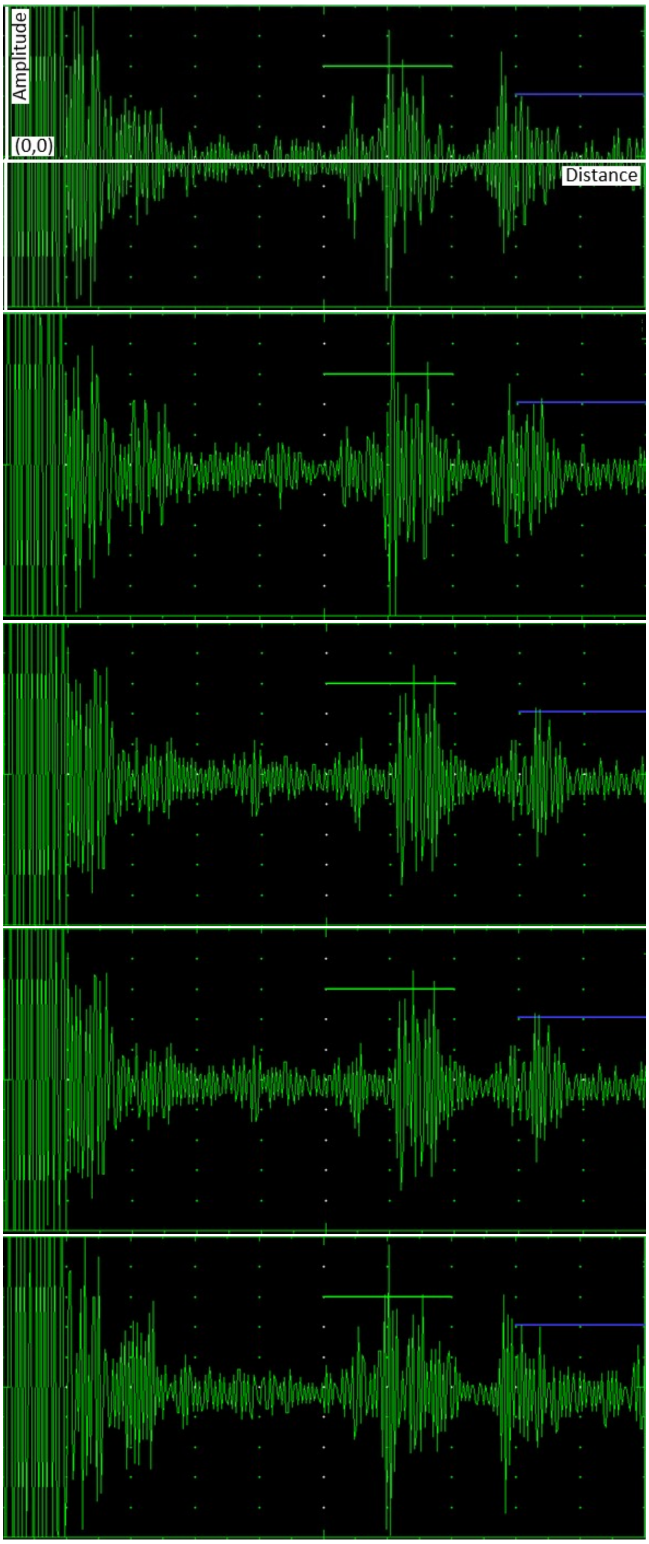

Fig. 14: Quarter bladder expanding: an attenuation signal from the posterior wall (second purple gate) and an attenuation signal from the anterior wall (first green gate). These signals were obtained in 10 minutes intervals.

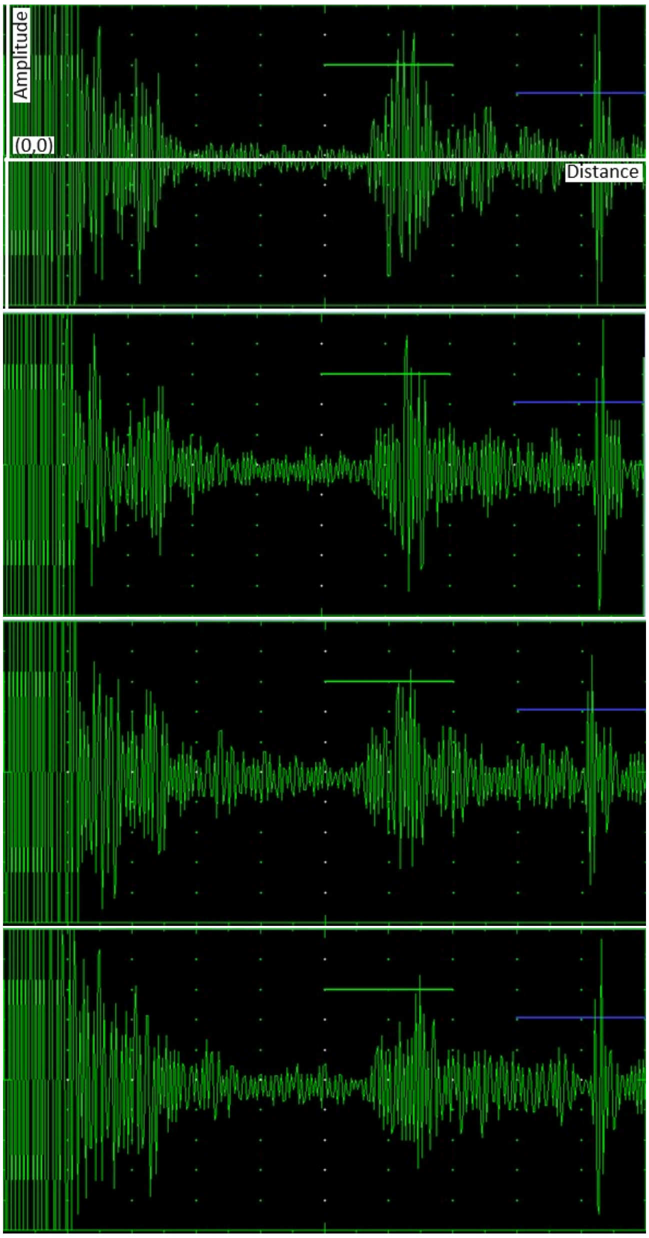

Fig. 15: Full bladder expanded considerably: an attenuation signal from the posterior wall (second purple gate) and an attenuation signal from the anterior wall (first green gate). These signals were obtained in 5 minutes intervals.
